# Artificial intelligence-guided discovery of a lead compound with antifolate-like activity against bacterial and human thymidylate synthases

**DOI:** 10.3389/fcimb.2026.1816809

**Published:** 2026-06-26

**Authors:** Yi-Lin Chen, Yi-Ming Chen, Yan Zou

**Affiliations:** 1 Department of Medical Oncology, Quzhou People's Hospital, The Quzhou Affiliated Hospital of Wenzhou Medical University, Quzhou, China; 2 Department of Traditional Chinese Gynecology, The First Affiliated Hospital of Naval Medical University, Shanghai, China

**Keywords:** AI-driven drug discovery, AlphaFold3, candidate cross-species ligand, colorectal cancer, phenotypic evaluation, target validation, thymidylate synthase (TYMS)

## Abstract

Thymidylate synthase (TYMS) is an established colorectal cancer (CRC) target. We evaluated whether conserved active-site architecture between human TYMS and the bacterial homolog TYSY KLEP7 could support early cross-species TYMS-axis lead discovery within the antifolate landscape. AlphaFold3, Chai-1 complex modeling, crystal-structure cross-validation, docking, ligand strain energy, MM-GBSA rescoring, and explicit-solvent MD were integrated to compare pocket states and prioritize antifolate-like Compound 8. Modeling indicated that Compound 8 preserves an acidic folate-groove anchoring motif while tolerating species- and ligand-induced pocket variation. Phenotypic assays showed dose-dependent inhibition of CRC cell viability with preliminary NCM460 selectivity, whereas antibacterial activity against *K. pneumoniae* was modest, with a MIC of 96 µM and bactericidal activity only at 4× MIC after 24 h. Target-axis assays supported TYMS/*thyA* engagement: siTYMS and AS-*thyA* reduced transcript levels, and Compound 8 inhibited crude-lysate TS activity with apparent AC_50_ values of 7.8 µM in HCT116 and 13.2 µM in *K. pneumoniae*. Overall, this work establishes an AI-enabled structure-to-phenotype framework and positions Compound 8 as an early antifolate-like TYMS-axis lead requiring purified-enzyme, ADME, safety, and antibacterial optimization studies.

## 1 Introduction

Colorectal cancer (CRC) remains a leading cause of global cancer-related mortality (Zhou et al., 2025), with thymidylate synthase (TYMS) serving as a cornerstone therapeutic target due to its essential role in DNA biosynthesis and repair. While 5-fluorouracil-based chemotherapies targeting TYMS have significantly improved survival rates, the clinical management of CRC can be complicated by opportunistic bacterial infections, such as *Klebsiella pneumoniae* (Longley et al., 2003; Yamauchi et al., 2023; Balboa-Beltrán et al., 2015). These observations provide clinical motivation for cautious cross-species target exploration, but the present study does not model secondary infection. Instead, cancer-cell and bacterial phenotypes are evaluated as separate *in vitro* readouts for early lead discovery. Traditionally, cancer and infection are treated as separate clinical entities; however, structure-guided lead discovery can generate hypotheses for coordinated target prioritization and chemical optimization, provided that antimicrobial potency, safety, and mechanism are independently validated.

Artificial intelligence (AI) and machine learning (ML) have reshaped pharmaceutical R&D by extending structure-based design beyond traditional high-throughput screening (Shao et al., 2022b; Kaur et al., 2025). Recent deep-learning tools, including AlphaFold3 and Chai-1, support protein-ligand complex prediction and cross-species structural modeling (Hou et al., 2025; Mathew et al., 2025). These tools can help identify conserved motifs across biological systems (Pathan et al., 2025). Given the structural conservation of catalytic pockets in human TYMS and bacterial homologs such as TYSY KLEP7, cross-species TYMS ligand discovery is a reasonable early lead-discovery question, although antibacterial translational claims require dedicated potency optimization and microbiological validation.

This rationale does not imply that folate-pathway conservation or antifolate dual pharmacology is new. Antifolate drugs already exploit conserved one-carbon metabolism across disease contexts: trimethoprim is a clinically important antibacterial DHFR inhibitor, pyrimethamine and related agents are antiprotozoal DHFR antifolates, raltitrexed is a direct folate-based TS inhibitor used in colorectal cancer, and pemetrexed/LY231514 is a multitargeted antifolate that inhibits several folate-dependent enzymes, including TS, DHFR, and GARFT (Kompis et al., 2005; Nzila, 2006; Jackman and Calvert, 1995; Zhou et al., 2013; Adjei, 2000). Prior medicinal-chemistry and computational studies have also pursued TS/DHFR dual-inhibitor antifolates (Gangjee et al., 2001; Arooj et al., 2013). Broader modern medicinal-chemistry strategies for TYMS inhibitor design have also recently been reviewed (Al-Karmalawy et al., 2026). Accordingly, the contribution of the present study is not the general concept of antifolate-based dual pharmacology, but a TYMS-axis, cross-species structural prioritization and validation workflow focused on human TYMS and the *K. pneumoniae* ThyA/TYSY KLEP7 homolog.

A continuing challenge is connecting computational predictions with experimental validation and realistic medicinal-chemistry positioning (Sreenivasan and Suresh, 2024). For antifolate-like scaffolds, known folate-pathway pharmacology must be distinguished from scaffold-specific behavior under species-specific thymidylate-synthase pocket constraints. This requires a framework that links structural biology, predictive modeling, target-axis assays, and cautious phenotype interpretation without overstating clinical translation.

In this study, we present an integrative AI-enabled structure-to-phenotype framework for prioritizing candidate cross-species TYMS ligands within this established antifolate context. By leveraging AlphaFold3-derived structural models and Chai-1 complex simulations, we performed a systematic analysis of the cross-species active-site architecture (Xu et al., 2024). Through a combination of conditional molecular generation and a four-model intersection workflow-incorporating molecular docking, ligand strain energy, MM-GBSA rescoring, and MD-based pose-stability assessment-we identified Compound 8 as an antifolate-like prioritized lead candidate. Our models suggest that this compound preserves a conserved acidic anchoring motif while adapting to subtle pocket differences; phenotypic assays then showed dose-dependent suppression of human CRC cell proliferation and modest *K. pneumoniae* antibacterial activity. Our findings highlight how structure-based hypotheses coupled with AI-driven design can nominate antifolate-like TYMS-axis candidates for next-step biochemical, microbiological, and medicinal-chemistry validation.

## 2 Methods

### 2.1 AF3 structure prediction and Chai-1 complex modeling

Structural predictions for the monomeric targets TYSY KLEP7 (UniProt A6TDG3) and TYSY HUMAN/TYMS HUMAN (UniProt P04818) were generated from the primary amino acid sequences using AlphaFold3 (AF3) (Abramson et al., 2024) with default settings for fold-level comparison. Experimentally determined human TYMS crystal structures were used for cross-validation rather than as primary modeling templates, because no matched experimental KLEP7 structures and no complete matched human/KLEP7 ligand-state set were available for all comparisons. Protein-ligand complex models were produced with the Chai-1 web interface (Chai Discovery Lab, https://lab.chaidiscovery.com) by submitting one protein sequence and one ligand SMILES per job. The five ligands were DB07577 (2, 4-diamino-5-phenyl-6-ethylpyrimidine; CCC1=C(C(=NC(=N1)N)N)C2=CC=CC=C2), DB08734 (6, 6-dimethyl-1-(3-(2, 4, 5-trichlorophenoxy)propoxy)-1, 6-dihydro-1, 3, 5-triazine-2, 4-diamine; CC1(N=C(N=C(N1OCCCOC2=CC(=C(C=C2Cl)Cl)Cl)N)N)C), DB03541 (10-propargyl-5, 8-dideazafolic acid; C#CCN(CC1=CC2=C(C=C1)N=C(NC2=O)N)C3=CC=C(C=C3)C(=O)N[C@@H](CCC(=O)O)C(=O)O), DB05308/ANX-510 (folitixorin; C1C2CN(CN2C3=C(N1)N=C(NC3=O)N)C4=CC=C(C=C4)C(=O)N[C@@H](CCC(=O)O)C(=O)O), and DB01101 (capecitabine; CCCCCOC(=O)NC1=NC(=O)N(C=C1F)[C@H]2[C@@H]([C@@H]([C@H](O2)C)O)O). Thus, ten Chai-1 complex-prediction jobs were run (two targets times five ligands). All options exposed in the Chai-1 web interface were kept at their default values, corresponding to the default multi-sample prediction mode (five output candidates per job); no user-supplied templates, MSA files, experimental restraints, covalent constraints, modified residues, or manual ligand poses were provided. For each target-ligand pair, the final model used downstream was the highest-ranked Chai-1 output that passed three predefined sanity checks: (i) an intact single protein chain covering the submitted UniProt sequence, (ii) ligand placement in the thymidylate-synthase folate/active-site groove rather than a terminal or solvent-exposed region, and (iii) no severe chain break or ligand-protein steric overlap that would prevent pocket extraction. PAE matrices and the per-residue confidence values stored in the exported coordinate B-factor fields were retained for reliability assessment. For experimental cross-validation of the human model, selected TYSY HUMAN complexes were sequence-aligned and Cα superposed onto the human TYMS crystal structures 1HZW and 1I00 from RCSB PDB, and global and ligand-proximal pocket RMSDs were calculated. The selected complex models were not manually edited before use as inputs for pocket characterization, docking validation, and molecular interaction analysis.

### 2.2 Sequence alignment and ESPript-like rendering

Global pairwise sequence alignment was conducted using the BioPython Align.PairwiseAligner module, employing a scoring scheme of 1.0 for matches, -0.5 for mismatches, and gap penalties of -10.0 for opening and -0.5 for extension (Cock et al., 2009). The aligned sequences for TYSY KLEP7 and TYSY HUMAN were subsequently exported in FASTA format. For structural annotation, secondary structure profiles were derived from PDB chain A using DSSP as the primary assignment tool. DSSP states were collapsed as follows: H, G, and I were assigned to helix (H); E and B were assigned to strand (E); and T, S, blank, or any other state was assigned to coil (C). If DSSP failed for a residue or the DSSP record was unavailable, backbone dihedral angles were calculated from the N, CA, and C atoms of residues i-1, i, and i+1. The fallback assignment was applied residue by residue: helix (H) was assigned when -100° ≤ *φ* ≤ -30° and -80° ≤ *ψ* ≤ -5°; strand (E) was assigned when -180° ≤ *φ* ≤ -40° and either 90° ≤ *ψ* ≤ 180° or -180° ≤ *ψ* ≤ -120°; all remaining residues, including terminal residues or residues with missing backbone atoms for which *φ* or *ψ* could not be computed, were assigned to coil (C). Contiguous H and E residues were merged only for graphical rendering, without additional smoothing or reassignment. To ensure precise spatial correspondence, the PDB-derived secondary structure string was mapped onto the target sequences via global alignment (Shao et al., 2022a). The final alignment was visualized in a high-resolution ESPript-like format, organized in blocks of 60 residues per line. This graphical representation integrated a stacked sequence-logo header, dual secondary structure tracks, depicted as red cylinders for α-helices and blue arrows for β-strands, and localized conservation highlighting. Specifically, fully conserved residues were marked in light blue with consensus dots, while physicochemically similar substitutions were highlighted in light gray with open dots, with residue indices provided at both termini for clarity (Yang et al., 2020).

### 2.3 Molecule generation and filtering

Candidate molecules were generated as a focused analog-enrichment library rather than as an exhaustive high-throughput docking collection. For each of the ten protein-ligand complex models, the bound ligand was identified with the Gemmi library from the ligand residue record, and a ligand-centered binding pocket was defined by selecting protein residues with any heavy atom within 6.0 Å of the ligand (Lu et al., 2024; Carpenter and Altman, 2024). The reference ligand for each complex was taken from the curated SMILES table when available; otherwise, it was derived from the bound coordinates using Open Babel or the RDKit open-source cheminformatics toolkit (version 2025.03.4; Landrum, 2025) and then standardized as a canonical, single-fragment SMILES. A maximum common substructure (MCS) mapping between the 2D reference ligand and the bound 3D ligand was used to define the atom correspondence for pose alignment (Wu et al., 2024). The maximum library size was capped at 250 unique canonical candidates per reference ligand before 3D scoring, defining a reproducible focused-screening limit before ETKDGv3 conformer generation, UFF relaxation, MCS-based alignment, pocket-interaction scoring, and docking/MM-GBSA follow-up across receptor contexts.

Two candidate-generation routes were implemented upstream. First, PubChem 2D-similarity retrieval was performed through the PubChem PUG REST fastsimilarity-2d endpoint using the reference ligand SMILES as the query, Threshold = 80 and MaxRecords = 250. Returned CIDs were converted to IsomericSMILES or CanonicalSMILES, canonicalized with RDKit, restricted to single-fragment molecules, and deduplicated while preserving source order. Second, an RDKit BRICS replacement route was available as a local fallback/enrichment procedure. BRICS denotes Breaking of Retrosynthetically Interesting Chemical Substructures, a rule-based fragmentation approach introduced for drug-like chemical fragment spaces (Degen et al., 2008). Here, RDKit BRICS refers to the rdkit.Chem.BRICS Python module in RDKit (Landrum, 2025). In this route, BRICS bonds in the reference ligand were identified with RDKit BRICS.FindBRICSBonds, one BRICS bond was fragmented at a time with dummy labels retained, the larger fragment was treated as the core, and compatible labeled R-groups mined from the default fragment seed set and source-derived seed molecules were rebuilt with BRICS.BRICSBuild. The BRICS settings were max-per-bond = 8, max-total = max(2 × max-candidates, 320) = 500 candidate attempts for max-candidates = 250, and random seed = 0. BRICS fragments themselves were not used directly as docked molecules; only sanitized, reconstructed full compounds represented as single-fragment canonical SMILES were allowed to enter the candidate list. The source-specific lists were then deduplicated and truncated to the 250-molecule cap, including the unchanged reference ligand.

Tanimoto similarity was used as a reference-ligand similarity gate, not as a within-library diversity cutoff. For every candidate, three similarities were calculated against the corresponding reference ligand: Morgan fingerprint Tanimoto similarity (radius 2, 2048 bits), RDKit topological fingerprint Tanimoto similarity, and MACCS-key Tanimoto similarity. The reported two-dimensional Tanimoto value is the maximum of these three values, and candidates were retained only when two-dimensional Tanimoto value ≥ 0.30. Thus, the 0.30 threshold removed molecules that were too dissimilar from the reference scaffold; it was not used to enforce dissimilarity among generated candidates. Additional upstream filters required QED ≥ 0.20 and no more than two Lipinski Rule-of-Five violations. RDKit descriptors were also calculated for developability context, including molecular weight, calculated logP, topological polar surface area (TPSA), hydrogen-bond donors and acceptors, rotatable bonds, and QED. In the final scored candidate tables used for this study, the 250-candidate cap and these filters yielded 168–248 retained molecules per complex. Across the ten complexes, 2100 scored records were retained: 2090 PubChem-similarity records and 10 reference-ligand records. No BRICS-primary candidates survived into the final scored tables in this dataset, indicating that the BRICS route was available but practically ineffective after the similarity, drug-likeness, and pose-quality filters used here.

For three-dimensional evaluation, retained candidates were generated via the RDKit ETKDGv3 algorithm and refined through UFF minimization (Wang et al., 2020). These candidates were then rigidly superimposed onto the reference pose using the MCS-derived atom correspondence. The final pocket-aware ranking was determined by calculating alignment root-mean-square deviation (RMSD) and assessing molecular interactions, including hydrogen bonds and steric clashes, against the pocket’s point cloud data.

### 2.4 Docking, MM-GBSA rescoring, strain energy, and interaction visualization

Molecular docking was performed with Schrödinger Glide in standard precision (SP) mode. The docking region was defined from the compound-binding region observed in the corresponding Chai-1 protein-ligand model. For each receptor, the Glide grid was centered on the Chai-1 ligand centroid; the inner grid box was 10 × 10 × 10 Å, and the outer box ranged from 25.36 to 27.82 Å across the four docking grids used for the representative human and bacterial 10-propargyl-5, 8-dideazafolic acid and ANX-510 receptor models. No positional constraints, interaction constraints, or receptor-flexibility settings were applied during Glide docking; the receptor was treated as rigid. Ligand van der Waals scaling used a scaling factor of 1.0 with a partial-charge cutoff of 0.25. For each ligand, ten docking poses were generated, and the pose with the best Glide score was retained for subsequent MM-GBSA rescoring, strain-energy calculation, and interaction inspection. No additional manual pose filtering or post-docking editing was applied before these downstream analyses.

Binding free energies were estimated for the selected best-scoring docking poses using Prime MM-GBSA in complex mode. The MM-GBSA calculations used the VSGB implicit-solvent model and the OPLS4 force field. Protein residues within 5 Å of the ligand were treated as the flexible region, while the remaining receptor atoms were kept fixed. The sampling method was Minimize; no molecular-dynamics trajectory frames or multiple-frame ensemble averages were used. Thus, each reported MM-GBSA value corresponds to a single minimized receptor-ligand complex generated from the best Glide SP pose for that ligand. Ligand strain energy was taken from the same Prime MM-GBSA output as the energetic penalty between the ligand geometry in the optimized complex and the optimized free-ligand state. Crystallographic waters were not retained as explicit energetic components in these MM-GBSA rescoring calculations, so the values are interpreted only as relative ranking estimates and not as absolute binding free energies. Protein-ligand interaction analysis was then performed directly with the Maestro Ligand Interaction module using its default settings, without additional user-defined distance or angle parameters. The resulting interaction diagrams were used to document hydrogen bonds, ionic contacts, aromatic interactions, and hydrophobic contacts within the binding pocket (Xu et al., 2024). Water-mediated contacts were assessed separately from explicit-solvent MD trajectories rather than from the implicit-solvent MM-GBSA term.

Four selected receptor-ligand complexes (TYSY HUMAN and TYSY KLEP7 in the 10-propargyl-5, 8-dideazafolic acid and ANX-510 pocket states) were simulated with Desmond. Systems were built with the S-OPLS force field, SPC water, a triclinic solvent box with a 10 Å buffer, neutralizing Na^+^ counterions, and 0.15 M NaCl. After the default Desmond relaxation protocol, production MD was run in the NPT ensemble at 300 K and 1.01325 bar, with a 9 Å nonbonded cutoff. The revised RMSD stability analysis used exported 0–1000 ns tables containing 1001 time points per trajectory. Ligand-protein contact diagrams were exported from the Desmond Simulation Interactions Diagram for the selected 0.00–100.00 ns trajectory using the display threshold for interactions occurring during more than 40.0% of the simulation time. Stability was assessed by protein-fit-on-protein Cα RMSD, ligand-fit-on-protein RMSD, ligand-fit-on-ligand RMSD, and recurrent ligand-protein contacts.

### 2.5 Computer-aided synthesis planning

Computer-aided synthesis planning was performed with RDKit BRICS, the rdkit.Chem.BRICS Python module implementing BRICS fragmentation and recombination utilities in the RDKit toolkit (Degen et al., 2008; Landrum, 2025), together with a complementary medicinal-chemistry feasibility assessment. For each target SMILES, BRICS-cleavable bonds were identified, cleaved, and converted into standardized fragment records for route visualization.

Dummy atoms introduced during cleavage were removed, and the resulting fragments were sanitized and standardized to canonical SMILES. In graph-based mode, fragmentations were expanded by breadth-first search with predefined limits on maximum depth and child nodes per expansion.

A representative route was generated by a greedy stepwise procedure that selected one BRICS bond per step, split the molecule into a recursively decomposed core and a side fragment, and favored cores retaining more internal BRICS bonds. Degenerate disconnections producing very small hydrocarbon fragments were penalized. The resulting route text was reviewed against medicinal-chemistry building-block classes, including protected glutamate esters, cyclopropanecarboxylate/acylating reagents, aryl electrophiles, heterocyclic amine partners, and standard coupling/deprotection reagents.

### 2.6 Cell viability assay (CCK-8)

Human colorectal cancer cell lines HCT116, SW480, and HT-29 and the normal colonic epithelial cell line NCM460 were maintained in their recommended complete media under humidified culture conditions at 37 °C and 5% CO_2_. Cells were seeded into 96-well plates at densities selected to keep vehicle-treated wells in the exponential-growth range during the assay window and were allowed to adhere overnight before Compound 8 treatment.

Compound 8 was dissolved in DMSO as a concentrated stock and diluted into complete medium immediately before use. The CCK-8 experiment used final Compound 8 concentrations of 0, 5, 10, 15, 20, 25, 30, 50, and 100 µM. The vehicle concentration was kept constant across all concentrations within each cell-line assay. After drug exposure, CCK-8 reagent was added according to the manufacturer’s colorimetric protocol, and absorbance was measured at 450 nm using a microplate reader.

For each cell line, raw OD_450_ values were normalized to the corresponding 0 µM vehicle mean, which was set to 100% viability. The source worksheet contains ten same-batch replicate wells per concentration (R1-R10); no day, passage, or independent-experiment metadata were present in the source file, so these replicate wells were used to summarize within-assay dispersion and were not treated as independent biological replicates. Viability is reported as mean ± SD of the same-batch wells. IC_50_ values were estimated with a four-parameter logistic model fitted to the normalized concentration-response data; R^2^ values and 95% confidence intervals are reported as curve-fitting diagnostics for this same-batch dataset. Selectivity index (SI) was calculated as IC_50_, NCM460 divided by IC_50_, CRC. Because NCM460 did not reach 50% inhibition within 0–100 µM, SI values were reported as conservative lower bounds using 100 µM as the NCM460 IC_50_ lower bound. Raw and processed records are provided in the accompanying CCK-8 source workbook and processed source tables.

### 2.7 Antibacterial endpoint, MIC, and time-kill assays against KLEP7

An initial antibacterial endpoint screen was performed as an exploratory CFU enumeration assay against *K. pneumoniae* KLEP7. Cultures were grown to the logarithmic phase, diluted to a standardized inoculum in fresh broth, and exposed to vehicle or Compound 8 under matched DMSO conditions. After exposure, samples were serially diluted in sterile saline or PBS, plated on non-selective agar, incubated, and enumerated as CFU/mL before log_10_ transformation. Each endpoint condition was represented by ten technical plate-count measurements in the screening dataset. These endpoint counts were used only as descriptive screening evidence and were not treated as independent biological replicates.

Broth microdilution was then performed to define the MIC of Compound 8 against *K. pneumoniae* ATCC 700721/MGH 78578. Compound 8 was tested at 0, 1, 2, 4, 8, 16, 24, 32, 48, 64, 96, and 128 µM with three assay replicates per concentration. OD_600_ was recorded after 20 h, visible growth was scored, and the MIC was prospectively defined as the lowest concentration at which all replicates had OD_600_ ≤ 0.10 and no visible growth. Time-kill assays were subsequently performed using the measured MIC of 96 µM. Vehicle, 0.5× MIC, 1× MIC, 2× MIC, and 4× MIC groups corresponded to 0, 48, 96, 192, and 384 µM Compound 8, respectively. Three assay replicates were collected at 0, 2, 4, 8, and 24 h, serially diluted, plated, and reported as log_10_ CFU/mL; the lower reporting limit was 1.7 log_10_ CFU/mL. Bactericidal activity was defined as a ≥3 log_10_ CFU/mL reduction from the starting inoculum. A separate MBC assay was not performed. Raw MIC and time-kill records are provided in the accompanying Q2 source tables.

### 2.8 *TYMS*/*thyA* transcript suppression and crude-lysate TS activity assays

TYMS transcript suppression in HCT116 cells was performed with three independent TYMS siRNA duplexes, siTYMS-H1, siTYMS-H2, and siTYMS-H3, targeting CDS nt 270–288, 447–465, and 741–759 of RefSeq TYMS transcript NM001071.4/UniProt P04818, respectively. A non-targeting siRNA was used as the negative control. TYMS expression was quantified by qPCR with the TYMS-qPCR primer pair and normalized to GAPDH-qPCR. In the bacterial system, *thyA* modulation was performed and reported as transcript suppression rather than siRNA knockdown. *K. pneumoniae* ATCC 700721/MGH 78578 was tested with three *thyA*-targeting antisense designs, AS-*thyA*-K1, AS-*thyA*-K2, and AS-*thyA*-K3, targeting CDS nt 146–166, 523–543, and 729–749 of CP000647.1 complement 3551066.3551860/UniProt A6TDG3. A scramble antisense oligo was used as the negative control, and *thyA*-qPCR was normalized to *rpoB*-qPCR. Three biological replicates and two technical qPCR wells per target/reference assay were recorded. Relative expression was calculated by the 2^(-ΔΔCt) method against the matched negative control, and suppression percentage was calculated as (1 - relative expression) × 100. Oligo sequences, primer sequences, Ct-level records, biological-replicate ΔΔCt calculations, and summaries are provided in the accompanying Q1 source tables.

Crude-lysate TS activity was measured in HCT116 and *K. pneumoniae* ATCC 700721 lysates normalized by total protein, with 20 µg protein per reaction in the raw records. Reactions used dUMP and reduced folate substrate conditions and were monitored by blank-corrected A_340_ slope or an equivalent TS activity readout after a 10 min preincubation. Compound 8 was tested directly at 0, 0.03, 0.1, 0.3, 1, 3, 10, 30, and 100 µM with three reaction replicates per dose. 5-FU was tested over the same concentration grid only under activation-compatible crude-lysate conditions and was interpreted as a functional TS-pathway readout rather than as direct purified-TS inhibition. FdUMP was included as a direct TS positive control at 0, 0.001, 0.003, 0.01, 0.03, 0.1, 0.3, 1, and 3 µM. Background-corrected slopes were normalized to vehicle control to calculate residual activity and inhibition percentage, and apparent crude-lysate AC_50_ values were assigned from the dose-response curves. Raw slope-level records and dose summaries are provided in the accompanying Q1 enzyme-activity source tables.

### 2.9 Statistical analysis

Statistical analyses were revised to distinguish same-batch or technical measurements from independent biological experiments. Unless stated otherwise, *n* values refer to the measurement unit specified for each assay. Same-batch CCK-8 replicate wells and endpoint CFU plate-count measurements were not treated as independent biological replicates. For exploratory CCK-8 and endpoint CFU comparisons, one-way ANOVA was used only as an omnibus assessment of concentration-associated differences, followed by prespecified treatment-versus-vehicle comparisons using Welch’s *t*-test with Holm correction for multiple comparisons. Because concentration is an ordered dose-response variable, potency was summarized with four-parameter logistic modeling rather than by interpreting all pairwise comparisons as independent biological effects. For the updated CCK-8 curves, R^2^ and 95% confidence intervals were calculated from the same-batch replicate-well data and are reported as curve-fitting diagnostics, not as biological-replicate inferential intervals. For CCK-8, MIC, and time-kill data, treatment-versus-vehicle effect sizes were calculated from available replicate-level data as Cohen’s d and small-sample-corrected Hedges’ g, with Welch confidence intervals for mean differences; the combined reanalysis is provided in the accompanying statistical reanalysis source table.

## 3 Results

### 3.1 Conserved catalytic architecture and pocket similarity between human TYMS and *K. pneumoniae* TYSY support a shared TYMS-axis scaffold

**Figure 1:**
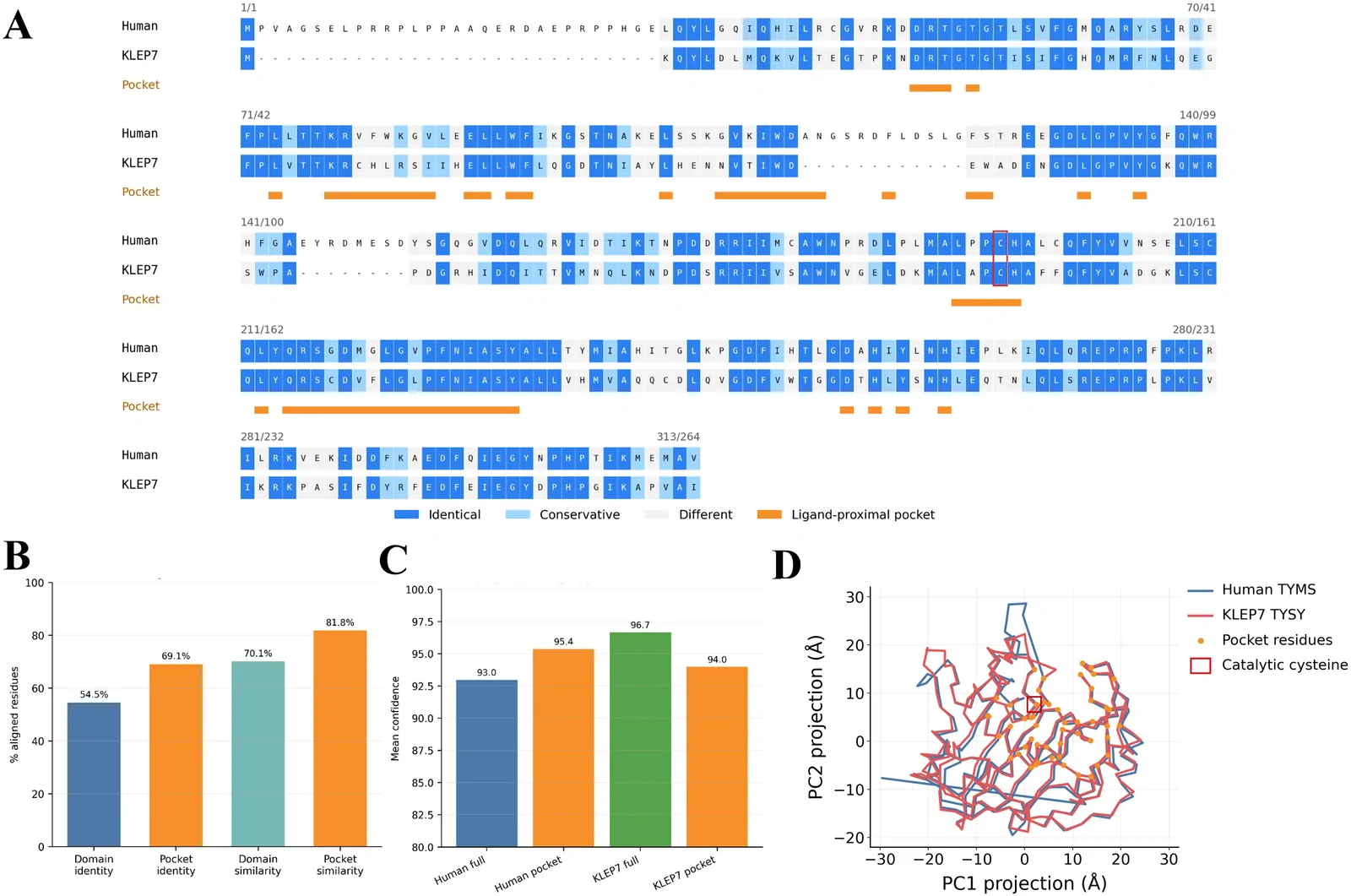
Sequence conservation and structural comparison of TYSY HUMAN and TYSY KLEP7. **(A)** Pairwise sequence alignment of TYSY HUMAN and TYSY KLEP7, with identical residues, conservative substitutions, divergent residues, and ligand-proximal pocket positions highlighted. The conserved catalytic cysteine pair is outlined by a red box. **(B)** Catalytic-domain versus ligand-proximal-pocket identity and physicochemical similarity. **(C)** Full-chain and pocket mean model confidence values for representative direct-antifolate pocket states. **(D)** Representative Cα superposition of human TYMS and KLEP7 TYSY models, with pocket residues highlighted. The corresponding catalytic cysteine position is outlined by a red box. The pocket is defined by residues within 8 Å of the ligand across the two direct-antifolate pocket-state models.

We used AlphaFold3/Chai-1 modeling for the comparative structural analysis because no complete matched experimental structure set was available for both human TYMS and the bacterial KLEP7 homolog across the same ligand-state comparisons; available human TYMS crystal structures were therefore used for cross-validation of the human models rather than as a substitute for the matched cross-species set. Since inhibiting human TYMS offers antitumor potential and bacterial TYMS homologs may provide antimicrobial starting points after optimization, we explored whether the catalytic core and ligand-binding pocket are sufficiently alike to permit a common scaffold or template for cross-species target-axis engagement. To investigate this possibility, we evaluated pairwise sequence conservation (**Figure 1A**), compared catalytic-domain and ligand-proximal-pocket conservation metrics (**Figure 1B**), assessed AlphaFold3/Chai-1 model confidence (**Figure 1C**), and performed three-dimensional Cα superposition to compare the core fold and functional-site alignment of both proteins (**Figure 1D**).

The pairwise sequence alignment in **Figure 1A** showed that the main sequence differences are located at the N terminus. Human TYMS contains a longer Pro/Glu-rich N-terminal extension, consistent with a species-specific flexible segment rather than a core catalytic element. Essential motifs such as RTGTGT and GDLGPVY occur at equivalent positions, and the catalytic cysteine is conserved, with Cys146 in the bacterial enzyme aligning to Cys195 in the human enzyme. The ligand-proximal pocket residues highlighted in **Figure 1A** further showed that the pocket is enriched for identical or conservatively substituted positions relative to the domain-wide alignment.

Quantitative conservation analysis in **Figure 1B** confirmed this pocket enrichment. Within the catalytic domain, the sequences align over approximately 264 positions, showing 54.5% identity and 70.1% physicochemical similarity. The ligand-proximal pocket is more conserved than the domain average: residues within 8 Å of the ligand across the two direct-antifolate pocket-state models contained 55 aligned shared positions, with 69.1% identity and 81.8% physicochemical similarity. The 10-propargyl-5, 8-dideazafolic acid and ANX-510 pocket states individually showed 71.2% identity/82.7% similarity and 62.8% identity/79.1% similarity, respectively.

The model-confidence summary in **Figure 1C** supported use of these models for active-site comparison. In representative direct-antifolate pocket-state models, the full-chain/pocket mean confidence scores were 92.9/95.1 for human TYMS and 96.6/93.7 for KLEP7 in the 10-propargyl-5, 8-dideazafolic acid state, and 93.1/95.6 for human TYMS and 96.7/94.2 for KLEP7 in the ANX-510 state. Higher uncertainty was concentrated in the human N-terminal extension and flexible loops, whereas pocket residues retained high confidence. The representative Cα superposition in **Figure 1D** further showed close alignment of the core secondary-structure architecture, with 264 aligned Cα pairs and a global Cα RMSD of 3.41 Å; structural divergence was limited mainly to flexible and solvent-exposed segments. Experimentally determined human TYMS structures were used as validation references rather than ignored: the TYSY HUMAN 10-propargyl-5, 8-dideazafolic acid model superposed onto human TYMS crystal structures 1HZW and 1I00 with global Cα RMSDs of 0.80 and 0.70 Å, respectively, and pocket Cα RMSDs of 0.60 and 0.66 Å, while the TYSY HUMAN ANX-510 model showed global RMSDs of 0.94 and 0.86 Å and pocket RMSDs of 0.78 and 0.77 Å. These low deviations support the predicted human active-site geometry, while the AF3/Chai-1 workflow provided a matched sequence-defined modeling set for the bacterial homolog and the paired ligand states needed for cross-species comparison.

### 3.2 DrugBank-derived human TYMS–associated scaffolds largely transfer to *K. pneumoniae* TYSY KLEP7 in Chai-1 complex models

Building on the observed similarity between species in catalytic machinery and pocket structure, we evaluated whether chemical scaffolds associated with human TYMS or its metabolic pathway can adopt similar complex conformations with the microbial version. This assessment was designed as a human-bacterial thymidylate-synthase structural-transferability analysis, not as a claim that antifolate pharmacology or folate-pathway dual activity is unprecedented. Five DrugBank entries linked to human TYMS were selected as reference molecules in **Table 1**. These entries represent two distinct classification levels. Specifically, DB07577 (2, 4 diamino 5 phenyl 6 ethylpyrimidine), DB08734 (6, 6 dimethyl triazine 2, 4 diamine), and DB03541 (10 propargyl 5, 8 dideazafolic acid) are categorized as experimental ligands with TYMS as their primary target. In contrast, DB05308 (folitixorin calcium) and DB01101 (capecitabine) are documented at the pathway level, where the former is investigational and the latter is approved or investigational. Both are connected to the TYMS pathway or the 5-fluorouracil mechanism. **Table 1** therefore offers two complementary types of chemical references including direct enzyme inhibitors and pathway-associated agents, allowing the study of conformational transferability within a biologically relevant context.

**Table 1:** DrugBank-recorded compounds.

DrugBank ID	Name	Drug type/status	Primary target(s) (as listed)
DB03541	10-Propargyl-5, 8-Dideazafolic Acid	Experimental	Ribosyldihydronicotinamide dehydrogenase [quinone]; Thymidylate synthase (recorded in some organism entries)
DB07577	2, 4-Diamino-5-phenyl-6-ethylpyrimidine	Experimental	Thymidylate synthase
DB08734	6, 6-Dimethyl-1-[3-(2, 4, 5-trichlorophenoxy)propoxy]-1, 6-dihydro-1, 3, 5-triazine-2, 4-diamine	Experimental	Thymidylate synthase
DB05308	Folitixorin calcium (ANX-510)	Investigational	Thymidylate synthase pathway (via 5-FU modulation)
DB01101	Capecitabine	Approved; Investigational	5-FU (tumor-activated prodrug), thymidylate synthase pathway

**Figure 2:**
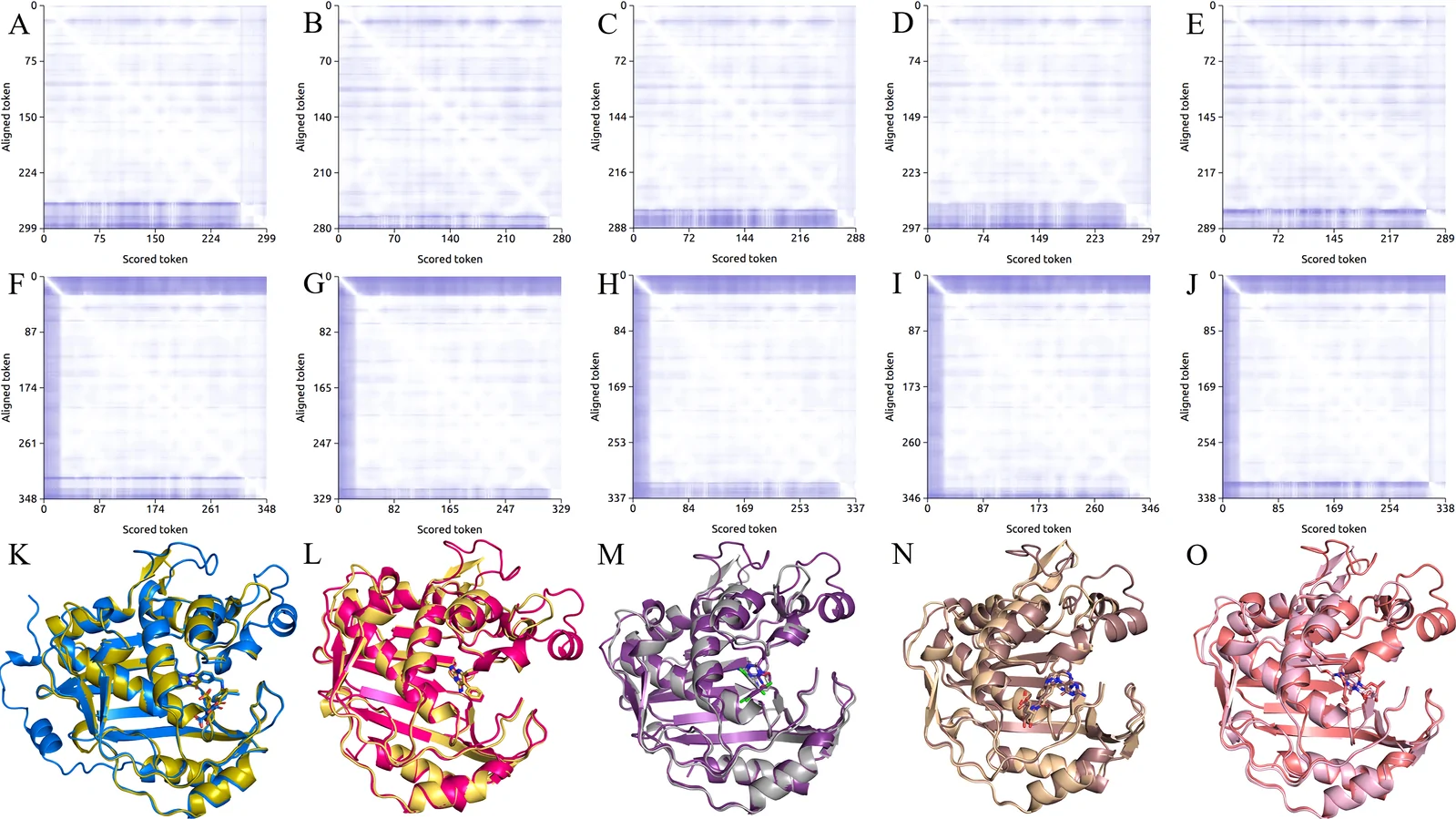
Prediction uncertainty and cross-species superposition of Chai-1–modeled TYSY–ligand complexes. **(A–E)** Predicted aligned error (PAE) matrices for Chai-1–modeled complexes between TYSY KLEP7 and five reference compounds. **(F–J)** PAE matrices for Chai-1–modeled complexes between TYSY HUMAN and the same five compounds. **(L–O)** Structural superposition of complexes for the same compound modeled with TYSY KLEP7 and TYSY HUMAN. Compounds are ordered as in **Table 1**: 10-propargyl-5, 8-dideazafolic acid; 2, 4-diamino-5-phenyl-6-ethylpyrimidine; 6, 6-dimethyl-1-(3-(2, 4, 5-trichlorophenoxy)propoxy)-1, 6-dihydro-1, 3, 5-triazine-2, 4-diamine; ANX-510; and capecitabine.

Chai-1 was utilized to generate complex models for TYSY KLEP7 and TYSY HUMAN with each of the five compounds, resulting in ten complexes. Predicted Aligned Error was applied to evaluate both the stability of the protein fold and the confidence in ligand placement as shown in **Figure 2A-J**. The error patterns for both proteins did not suggest domain level fragmentation, confirming that global fold predictions remain stable when the ligand is integrated. Consistent with typical small molecule complex predictions, band like features were observed near the edges of the matrix and terminal regions, indicating higher uncertainty in ligand positioning relative to the protein compared to the internal protein geometry. For the human enzyme, heterogeneous signals were frequently linked to the N-terminal region, which aligns with its extended N terminus and suggests that this part contributes flexibility without affecting the core pocket structure. These assessments confirm that the complex models are suitable for further use because uncertainty is concentrated in peripheral flexible regions rather than the catalytic core.

To evaluate pocket permissiveness across-species, the complexes generated for each compound against the human and bacterial proteins were superposed and compared in **Figure 2L-O**. Four of the five compounds demonstrated strong agreement in protein-core alignment and ligand localization within the pocket. Quantitative superposition supported this interpretation: after homologous Cα alignment, the ligand RMSDs for 10-propargyl-5, 8-dideazafolic acid, 2, 4-diamino-5-phenyl-6-ethylpyrimidine, 6, 6-dimethyl triazine diamine, and ANX-510 were 1.494, 1.378, 2.146, and 2.206 Å, respectively. The corresponding binding-pocket protein Cα RMSDs, calculated for residues within 8 Å of the ligand, were 2.604, 3.072, 1.844, and 2.631 Å, respectively. Capecitabine was the clear outlier, with a ligand RMSD of 5.770 Å and a pocket Cα RMSD of 2.350 Å, confirming a substantial pose shift between the two enzymes. Overall, these findings indicate that most human-associated scaffolds can form comparable complex structures across both enzyme types. Consequently, the Chai-1 complexes provide standardized starting conformations for subsequent docking, pose rescoring, and binding-energy estimation via MM-GBSA, which helps prioritize candidate inhibitory scaffolds for cross-species testing.

### 3.3 Complex-conditioned molecular generation identifies transferable chemical space and convergent cross-model hits

**Figure 3:**
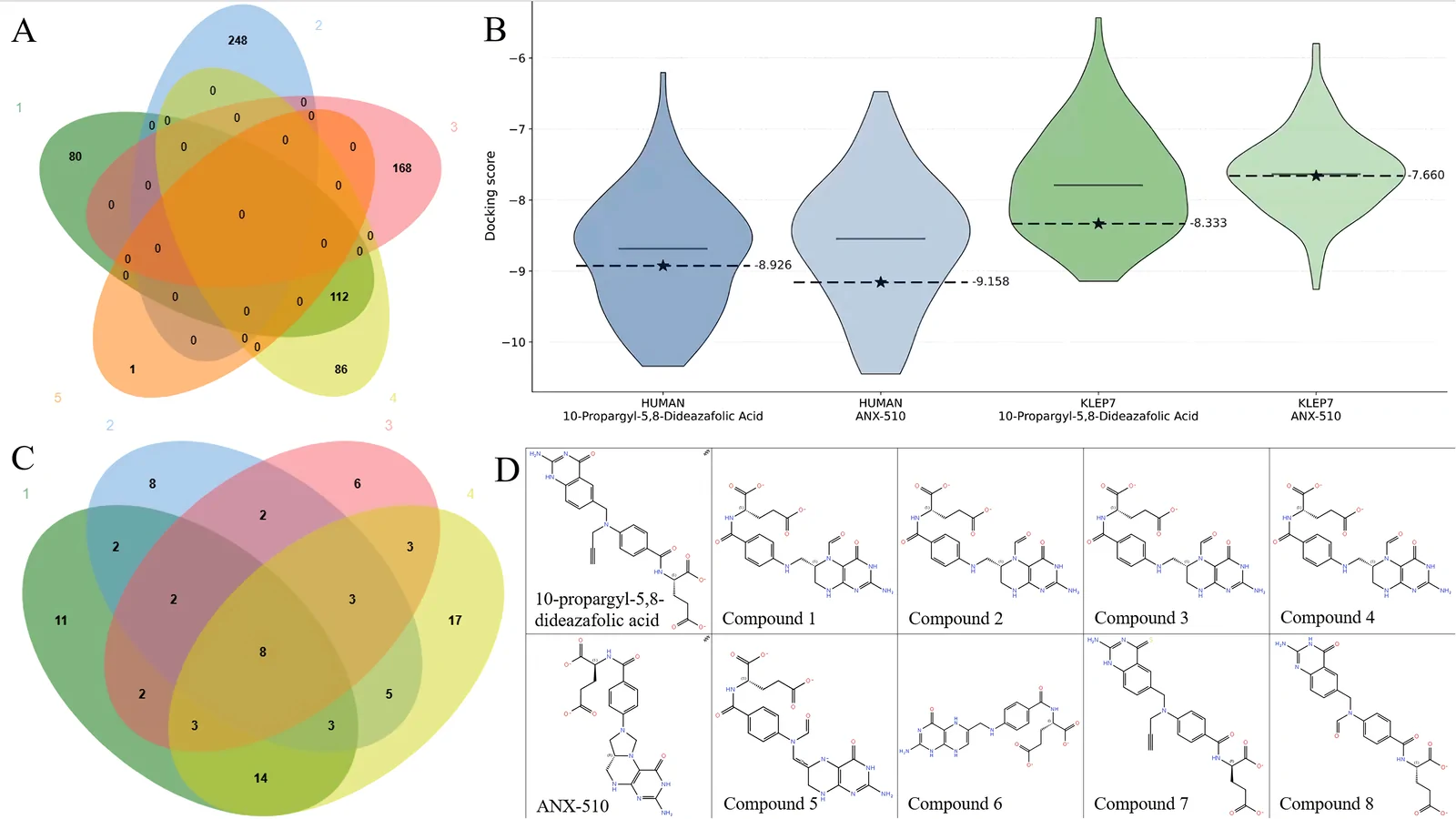
Complex-conditioned molecular generation and cross-model selection. **(A)** Venn diagram showing five generated compound sets grouped by the reference ligand used for complex-conditioned generation (based on the 10 complex models). Groups 1–5 correspond to 10-propargyl-5, 8-dideazafolic acid; 2, 4-diamino-5-phenyl-6-ethylpyrimidine; 6, 6-dimethyl-1-(3-(2, 4, 5-trichlorophenoxy)propoxy)-1, 6-dihydro-1, 3, 5-triazine-2, 4-diamine; folitixorin calcium (ANX-510); and capecitabine. **(B)** Violin plots of docking score distributions for the 112 shared generated molecules docked to four receptor models; dashed lines indicate the docking scores of the corresponding reference compounds for each model. **(C)** Venn diagram of compounds with docking scores better than the corresponding reference compound in each of the four docking runs. Groups 1–4 correspond to TYSY HUMAN–10-propargyl-5, 8-dideazafolic acid; TYSY HUMAN–folitixorin calcium (ANX-510); TYSY KLEP7–10-propargyl-5, 8-dideazafolic acid; and TYSY KLEP7–folitixorin calcium (ANX-510). **(D)** 2D structures of the two reference compounds (left; 10-propargyl-5, 8-dideazafolic acid and folitixorin calcium (ANX-510)) and the eight selected generated candidates (right), compounds 1–4 are stereoisomers of each other.

To explore pocket transferability within chemical space, structure conditioned molecular generation was conducted using ten protein ligand complex models as constraints. We evaluated whether pocket states induced by specific reference ligands could generate candidate sets applicable to both human and bacterial enzyme contexts as illustrated in **Figure 3A**.

Each reference compound was associated with two complexes to form a paired conditioning set, resulting in five generated libraries analyzed via Venn diagrams in **Figure 3A**. The specific molecule counts were 80 for 10 propargyl 5, 8 dideazafolic acid, 248 for 2, 4 diamino 5 phenyl 6 ethylpyrimidine, 168 for the triazine derivative, 86 for folitixorin calcium, and 1 for capecitabine. A significant overlap was exclusively found between the libraries conditioned by 10 propargyl 5, 8 dideazafolic acid and folitixorin calcium, which shared 112 molecules. Other combinations showed no meaningful convergence. This result suggests that these two reference ligands guide the generation process toward a chemically viable region that is reusable across multiple pocket configurations, while the capecitabine conditioned space appears highly specific with minimal commonality.

The 112 shared molecules were defined as a representative cross-species transferable set and docked against four receptor models including human and bacterial TYMS conditioned by either 10 propargyl 5, 8 dideazafolic acid or folitixorin calcium. The distribution of docking scores was displayed in violin plots in **Figure 3B**. Dashed lines indicated the docking thresholds for the reference compounds at minus 8.926, minus 9.158, minus 8.333, and minus 7.660 respectively. Variations in score centers and dispersion across the receptors demonstrate that species origin and specific pocket states influence binding compatibility. These benchmarks provided a consistent method for selection across different models.

Molecules that exceeded the reference thresholds for each docking run were extracted and compared using Venn analysis in **Figure 3C**. This process revealed a high level of convergence where only eight compounds outperformed all four reference benchmarks. These candidates represent molecules with consistent binding advantages across both enzyme species and both pocket contexts. Consequently, they were prioritized for rigorous computational validation such as pose inspection and binding free energy estimation.

To allow for direct examination of chemical features, 2D structures of the two reference ligands and the eight selected hits were compiled in **Figure 3D**. Notably, compounds 1 through 4 are stereoisomers. This visualization serves as a foundation for examining shared pharmacophoric elements and substituent modularity while establishing an explicit input set for more stringent energy filtering and stability tests.

### 3.4 Tri-metric cross-model profiling refines priority dual-target candidates beyond docking performance

**Figure 4:**
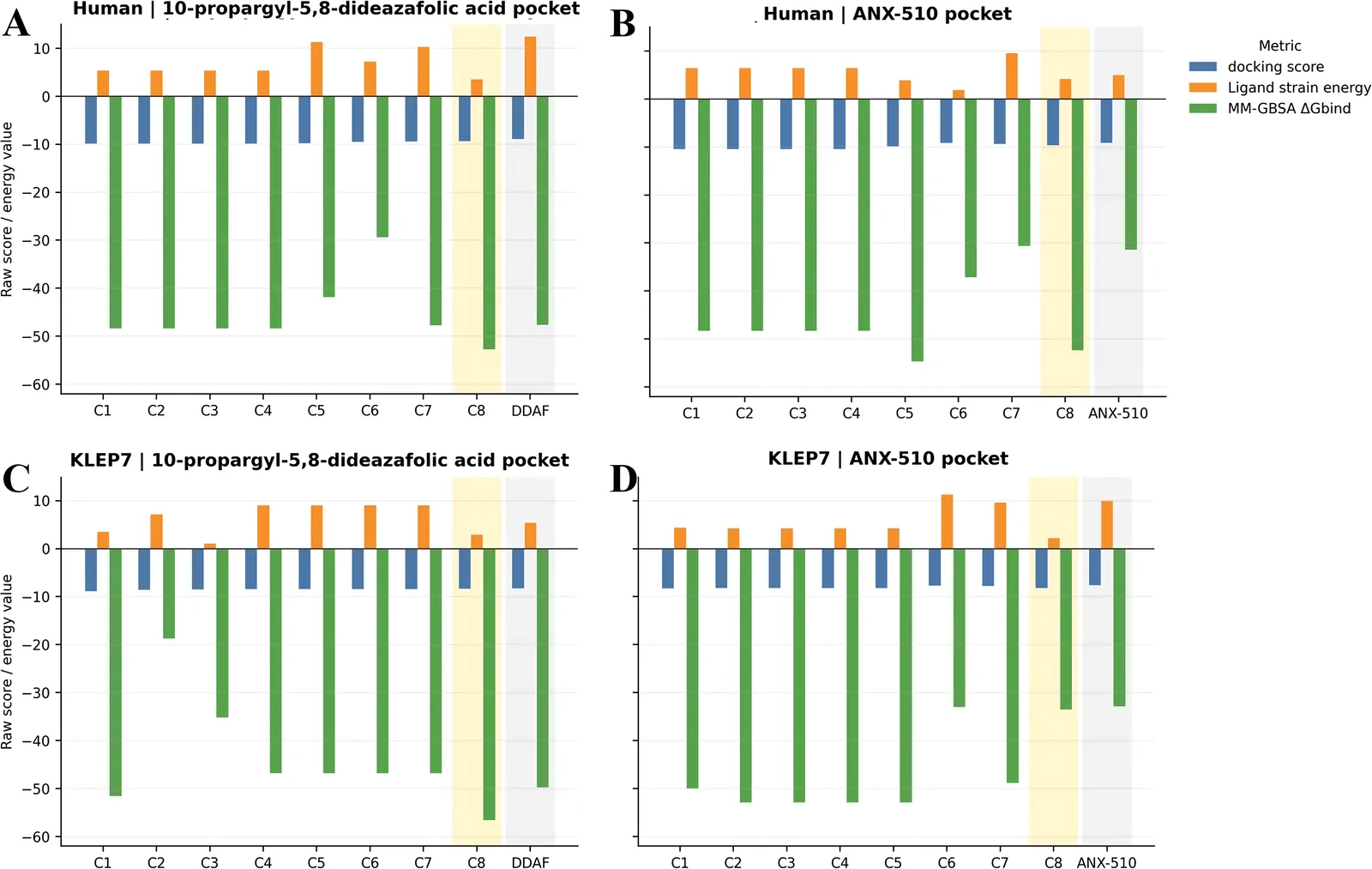
Tri-metric scoring of eight candidates across four induced-pocket receptor models. Panels summarize, for each compound within the same receptor model, the docking score, ligand strain energy, and MM-GBSA ΔGbind; a single shared color legend is placed to the right of panel **(B)** C1-C8 correspond to Compounds 1-8, with Compound 8 highlighted and the matched reference ligand shaded separately. **(A)** TYSY HUMAN induced-pocket model derived from the TYSY HUMAN-10-propargyl-5, 8-dideazafolic acid complex. **(B)** TYSY HUMAN induced-pocket model derived from the TYSY HUMAN-folitixorin calcium (ANX-510) complex. **(C)** TYSY KLEP7 induced-pocket model derived from the TYSY KLEP7-10-propargyl-5, 8-dideazafolic acid complex. **(D)** TYSY KLEP7 induced-pocket model derived from the TYSY KLEP7-folitixorin calcium (ANX-510) complex. The full numeric matrix is provided in the accompanying this image source matrix.

To further refine the selection of the eight candidates that outperformed reference docking scores across all four receptor models, a parallel evaluation using three distinct metrics was conducted for each candidate and its corresponding reference within the four induced pocket environments. These metrics included docking scores for geometric fit, ligand strain energy for conformational penalty, and MM-GBSA binding free energy as shown in **Figure 4A-D**, with detailed scoring results provided in **Supplementary Table 1** and the accompanying **Figure 4** source matrix. While candidates generally achieved superior docking scores compared to the references, significant stratification appeared in strain energy and MM-GBSA values. This demonstrates that improved docking does not always result in better binding energetics and highlights the importance of multi-parameter integration for reliable ranking.

In the human TYMS model induced by 10 propargyl 5, 8 dideazafolic acid (**Figure 4A**), Compounds 1 through 4 achieved the highest docking scores of negative 9.879, combined with a low strain energy of 5.421 and improved MM-GBSA relative to the reference. Compound 8 demonstrated the most significant overall advantage by pairing a low strain energy of 3.602 with the most favorable MM-GBSA of negative 52.76. In contrast, while Compounds 5 and 6 showed better docking than the reference, their MM-GBSA values were considerably weaker at negative 41.83 and negative 29.35, suggesting their geometric fit lacks strong energetic support.

In the human TYMS model induced by folitixorin calcium (**Figure 4B**), candidate separation was primarily determined by MM-GBSA results. Compounds 5 and 6 attained the top tier with improved docking, lower strain energy, and the highest MM-GBSA gains, significantly exceeding the reference. Compounds 1 through 4 reached top docking scores and substantially improved MM-GBSA, though their strain energy was slightly higher than the reference. Compound 8 exhibited the lowest strain energy but only moderate MM-GBSA gains, suggesting its advantage here is based on reduced conformational costs.

In the bacterial enzyme model induced by 10 propargyl 5, 8 dideazafolic acid (**Figure 4C**), all candidates surpassed the reference docking score, but MM-GBSA values varied greatly. Compound 8 was again the strongest, combining low strain energy with a superior MM-GBSA of negative 56.59. Compound 1 also outperformed the reference across all three metrics. Conversely, Compounds 2 and 3 showed very weak MM-GBSA values, and other candidates failed to provide consistent energetic support.

In the bacterial enzyme model induced by folitixorin calcium (**Figure 4D**), docking scores were generally better than the reference, but ligand strain energy became the main discriminator. No single candidate was optimal across all three metrics in this pocket state. Compound 8 retained favorable docking and comparatively strong MM-GBSA support, but its profile was less balanced than in the 10-propargyl-5, 8-dideazafolic acid-conditioned bacterial model, indicating that the ANX-510-conditioned KLEP7 pocket imposes a context-dependent conformational cost on antifolate-like scaffolds. Compounds 2 through 5 formed the most strain-efficient group in this specific pocket state.

Overall, this cross-model assessment identifies priority candidates that combine favorable docking, manageable strain energy, and favorable MM-GBSA across different pocket contexts. Compound 8 was particularly strong in both human and bacterial models induced by 10-propargyl-5, 8-dideazafolic acid and was retained for explicit MD stability validation because it preserved favorable energetics in the conserved thymidylate-synthase pocket while exposing a clear optimization liability in the KLEP7 ANX-510 pocket. Compounds 2 through 5 showed the greatest strain efficiency in the bacterial model with folitixorin calcium. Together with the strong performance of Compounds 5 and 6 in the human model, these molecules represent a refined set for focused follow-up studies, including binding-mode inspection and molecular-dynamics stability assessments.

### 3.5 Interaction-network plasticity enables compound 8 to accommodate conserved anchors across human and bacterial TYMS pockets

**Figure 5:**
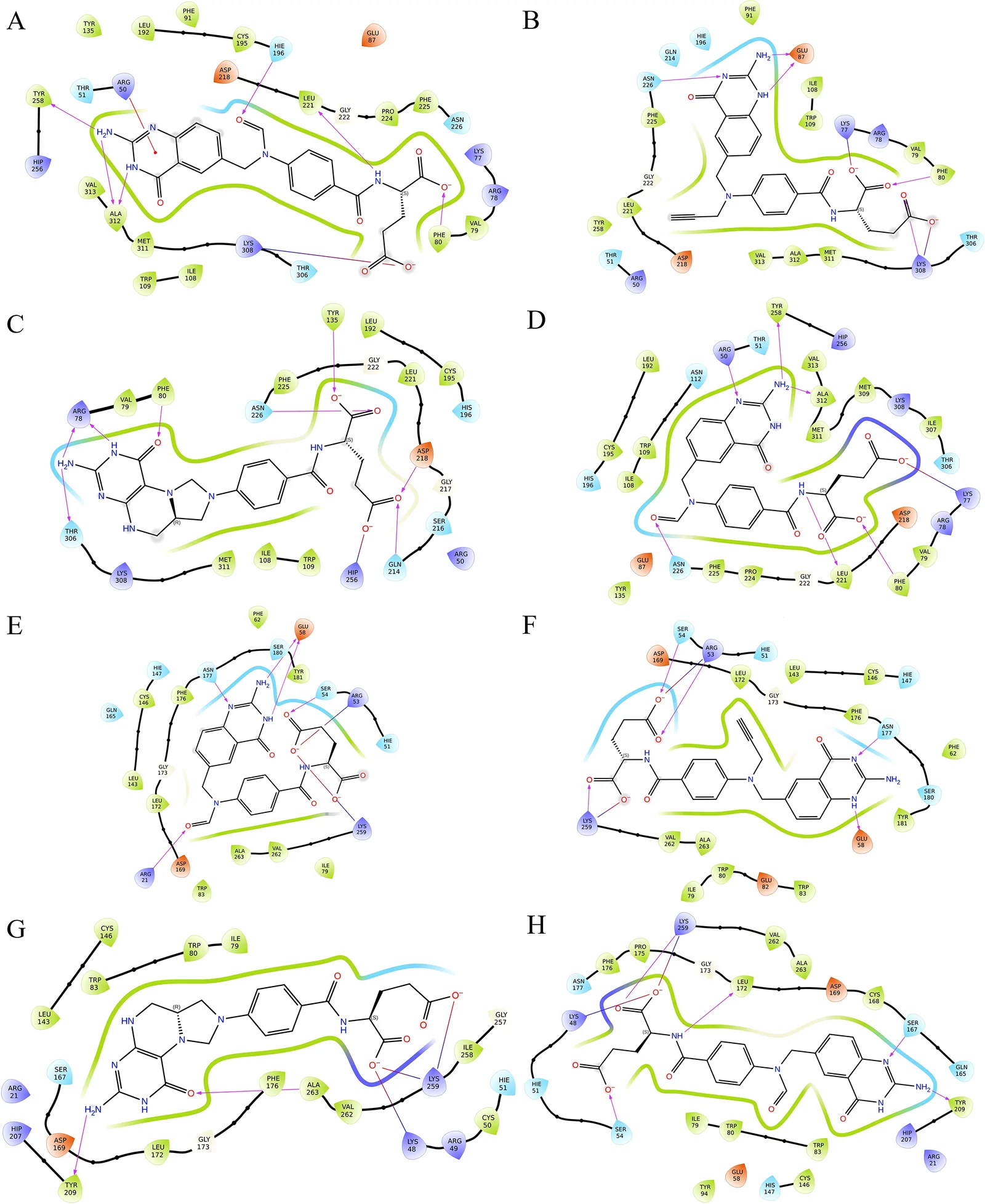
Comparative binding interaction patterns of Compound 8 and reference ligands in TYSY HUMAN and TYSY KLEP7 (Maestro 2D ligand interaction diagrams). **(A)** Compound 8 bound in the 10-propargyl-5, 8-dideazafolic acid pocket of TYSY HUMAN. **(B)** 10-propargyl-5, 8-dideazafolic acid bound to TYSY HUMAN. **(C)** Compound 8 bound in the ANX-510 pocket of TYSY HUMAN. **(D)** ANX-510 bound to TYSY HUMAN. **(E)** Compound 8 bound in the 10-propargyl-5, 8-dideazafolic acid pocket of TYSY KLEP7. **(F)** 10-propargyl-5, 8-dideazafolic acid bound to TYSY KLEP7. **(G)** Compound 8 bound in the ANX-510 pocket of TYSY KLEP7. **(H)** ANX-510 bound to TYSY KLEP7. The original high-resolution figure file will be supplied separately to prevent compression-related loss of readability; quantitative Compound 8 pose RMSDs are summarized in Section 3.5, while the revised 1000 ns RMSD traces and >40% ligand-protein contact diagrams are summarized in Section 3.6, **Figures 6** and **7**, the accompanying **Figures 6** and **7** source tables and source mapping files.

A systematic comparison of molecular interactions was conducted between Compound 8 and two reference molecules, 10-propargyl-5, 8-dideazafolic acid and ANX-510, within the binding sites of both human TYMS and the KLEP7 bacterial homolog as depicted in **Figure 5**. Compound 8 was interpreted as antifolate-like rather than as a wholly new chemotype. It shares the classical TYMS ligand logic of a heteroaromatic polar head, a para-benzoyl linker region, and a dicarboxylated acidic tail capable of engaging the folate-binding groove. This relationship makes raltitrexed, pemetrexed, and 10-propargyl-5, 8-dideazafolic acid closer structural comparators than trimethoprim or pyrimethamine, which are primarily DHFR-directed nonclassical antifolates. Despite the shared antifolate logic, Compound 8 demonstrates a more flexible binding orientation and compensatory interaction schemes compared with the reference ligands, suggesting a more plastic binding mode across the modeled TYMS/ThyA pocket states. A qualitative positioning summary is provided in the accompanying Compound 8 antifolate-positioning source table. To avoid loss of detail caused by manuscript-level image compression, the original high-resolution **Figure 5** file will be provided as a separate production/source figure.

In the human enzyme diagrams shown in **Figure 5A-D** and the MD contact-frequency summary, Compound 8 retained the expected acidic-tail recognition pattern but redistributed its polar contacts across pocket states. In the 10-propargyl-5, 8-dideazafolic acid-conditioned model, persistent contacts involved Asn112 (78.5% any-contact occupancy), Lys77 (67.7%), Asn226 (59.3%), Arg78 (46.4%), Phe80 (42.5%), and Lys308 (29.4%). In the ANX-510-conditioned model, the most persistent contacts shifted toward Asn226 (90.6%), Arg78 (86.1%), Phe80 (85.8%), Asn112 (54.4%), Met309 (40.6%), and Lys308 (36.1%). These patterns indicate that Compound 8 preserves the conserved folate-groove anchoring logic while using a reorganized polar network rather than exactly copying the interaction pattern of a single reference inhibitor.

Within the bacterial enzyme diagrams shown in **Figure 5E-H** and the MD contact-frequency summary, Compound 8 also preserved a dual anchoring pattern but with pocket-dependent contact redistribution. In the 10-propargyl-5, 8-dideazafolic acid-conditioned model, the dominant contacts were Glu58 (95.5%), Asn177 (79.2%), Ala260 (56.9%), Lys259 (49.3%), Ile258 (40.3%), and Lys48 (36.4%). In the ANX-510-conditioned model, contact persistence shifted toward Lys48 (82.5%), Ile258 (74.8%), Ala260 (65.1%), His207 (63.0%), Asn177 (44.0%), and Lys259 (40.3%). Thus, the bacterial models support an adaptable interaction network in which Compound 8 retains conserved basic-tail and head-region contacts while accepting different local interaction partners across induced pocket states.

Two main themes are evident from these results. First, the electrostatic stabilization of the acidic tail by a primary basic residue, specifically Lys308 in human and Lys259 in the bacterial enzyme, serves as a universal foundation for ligand binding. Second, Compound 8 preserves this essential tail anchor while utilizing adaptive strategies at the head and linker sites. It employs additional hydrogen bonds and backbone contacts to offset changes in electrostatic dependencies. This reconfigurable network likely explains the ability of the compound to adapt across different species and pocket environments. Detailed metrics including RMSD and interaction statistics are available in the supplementary documentation.

The final Compound 8 poses were also compared quantitatively using the TYSY HUMAN 10-propargyl-5, 8-dideazafolic acid pocket model as the reference. The ligand RMSDs for Compound 8 in the TYSY HUMAN ANX-510, TYSY KLEP7 10-propargyl-5, 8-dideazafolic acid, and TYSY KLEP7 ANX-510 pocket models were 1.629, 2.005, and 2.043 Å, respectively. The corresponding binding-pocket protein Cα RMSDs were 0.726, 2.382, and 2.411 Å, respectively, while the global Cα RMSDs were 3.158, 3.428, and 3.391 Å. These values indicate conserved pocket localization with moderate, pocket-dependent ligand reorientation rather than wholesale displacement of the binding pose.

Because static interaction diagrams cannot establish pose persistence, the four prioritized Compound 8 complexes were advanced to explicit-solvent Desmond molecular dynamics. Trajectory-level RMSD trends and contact persistence are reported in Section 3.6.

### 3.6 Explicit-solvent molecular dynamics supports persistent but adaptive compound 8 binding

**Figure 6:**
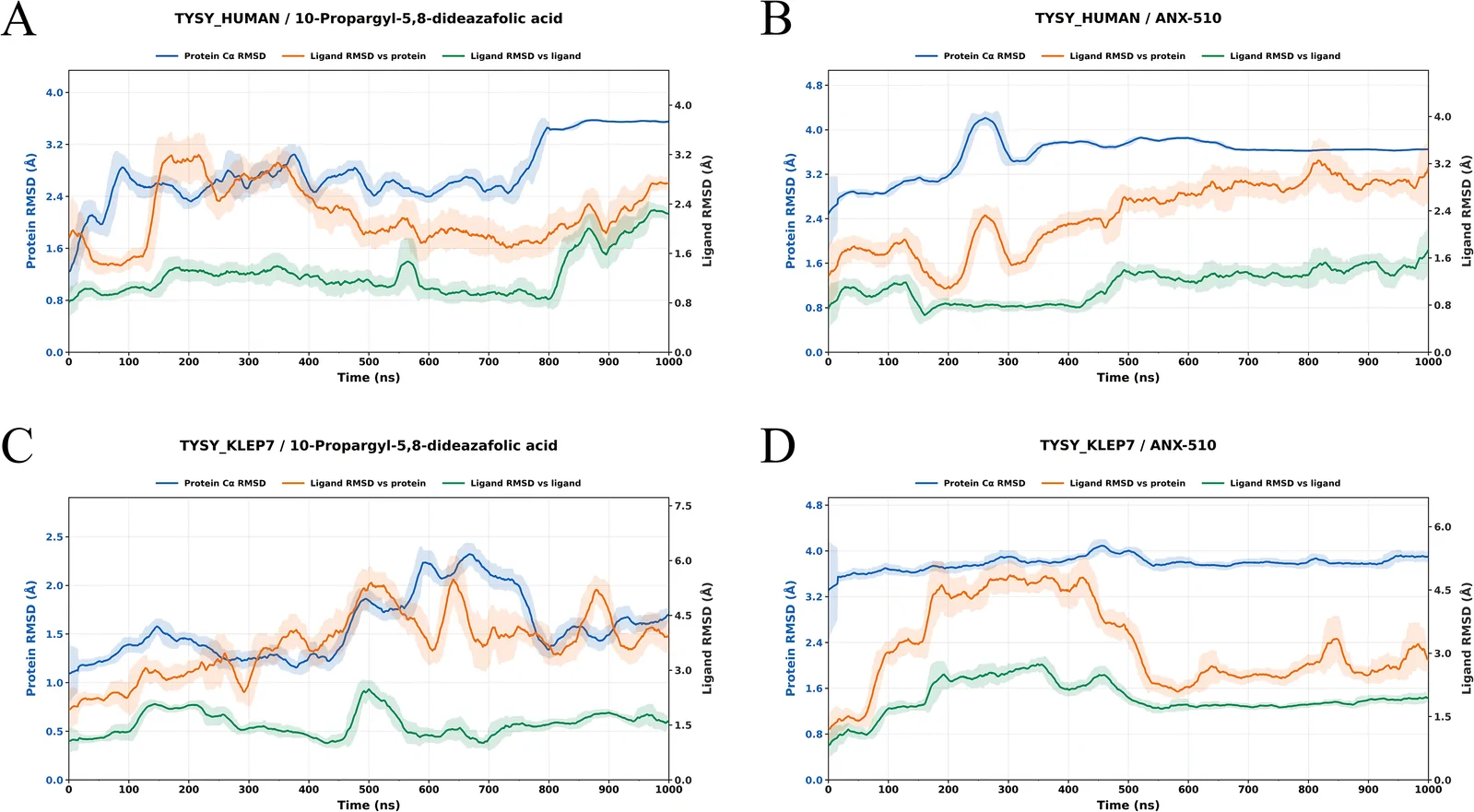
1000 ns RMSD stability of Compound 8 in four TYSY receptor contexts. **(A-D)** RMSD traces from 0–1000 ns tables for Compound 8 bound to TYSY HUMAN or TYSY KLEP7 in 10-propargyl-5, 8-dideazafolic acid- or ANX-510-conditioned pockets. The blue trace shows protein-fit-on-protein Cα RMSD on the left axis, and the orange and green traces show ligand-fit-on-protein and ligand-fit-on-ligand RMSD on the right axis, respectively. Shaded bands indicate local trajectory variation around the smoothed trend lines. Source values are provided in the accompanying this image RMSD source table and source mapping file.

**Figure 7:**
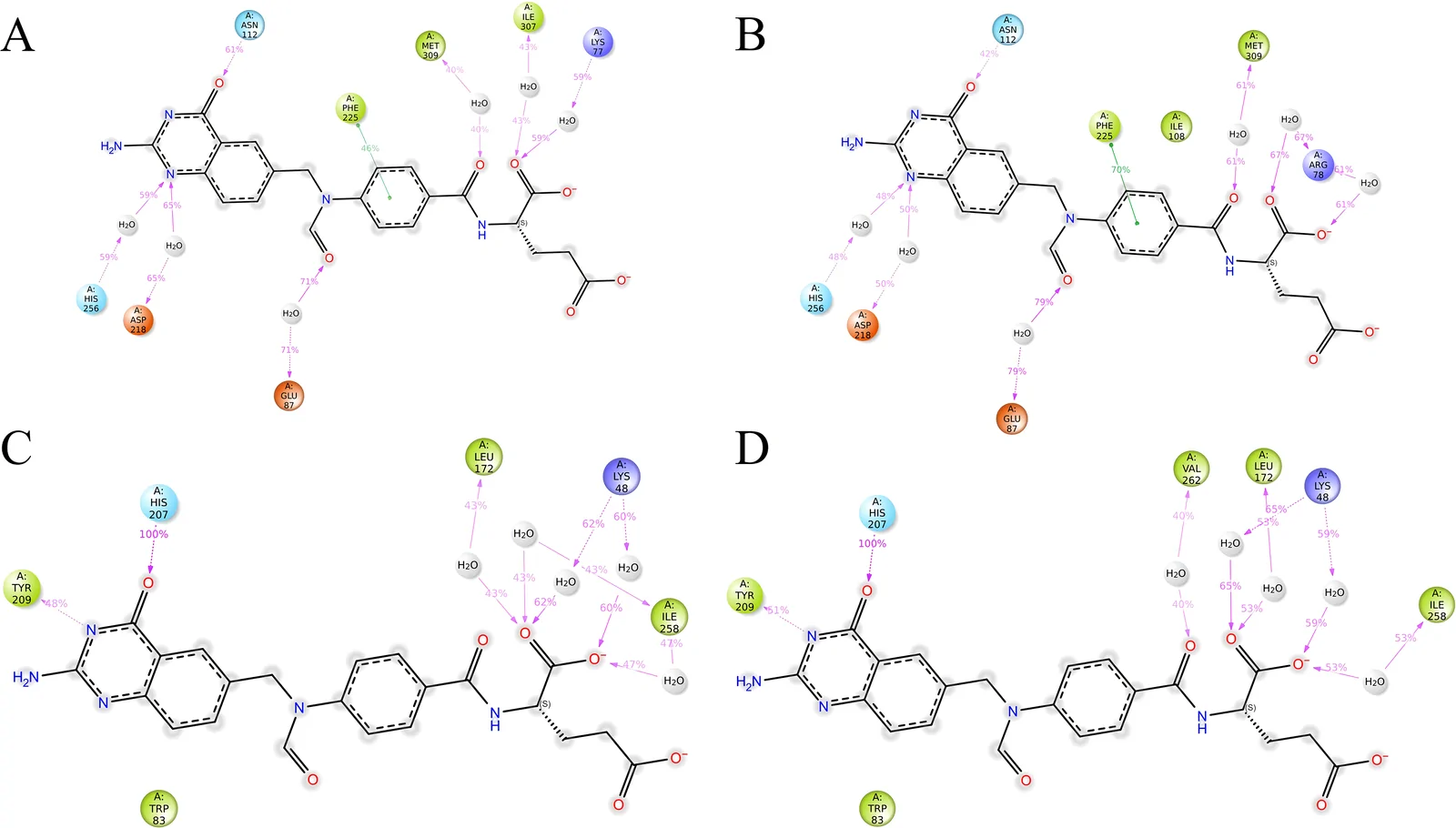
Recurrent ligand-protein contacts of Compound 8 in four TYSY receptor contexts. **(A-D)** Ligand-Protein Contacts diagrams showing interactions that occur during more than 40.0% of the simulation time in the selected trajectory from 0.00 through 100.00 ns. Panels A-D correspond to the same receptor/pocket order as **Figure 6**: TYSY HUMAN 10-propargyl-5, 8-dideazafolic acid, TYSY HUMAN ANX-510, TYSY KLEP7 10-propargyl-5, 8-dideazafolic acid, and TYSY KLEP7 ANX-510. Source values and file mapping are provided in the accompanying this image contact source table and source mapping file.

The revised MD stability analysis used the four 0–1000 ns RMSD tables and ligand-protein contact diagrams provided with the **Figure 6** and **Figure 7** source materials to test whether the prioritized Compound 8 binding poses remained stable in explicit solvent. The analyzed systems comprised TYSY HUMAN and TYSY KLEP7 in the 10-propargyl-5, 8-dideazafolic acid and ANX-510 induced-pocket states. Each RMSD table contained 1001 time points from 0 to 1000 ns for protein-fit-on-protein Cα RMSD, ligand-fit-on-protein RMSD, and ligand-fit-on-ligand RMSD.

Inspection of the 0–1000 ns RMSD traces revealed pocket-dependent but generally persistent binding behavior across the four simulated receptor contexts. In the TYSY HUMAN 10-propargyl-5, 8-dideazafolic acid system, the protein Cα trace rose during early equilibration and then remained on a low-to-moderate plateau; the ligand-fit-on-protein trace stayed close to the binding pocket with only mild late upward drift, while the ligand-fit-on-ligand trace remained compact, supporting retention of the docked ligand shape. In the TYSY HUMAN ANX-510 system, the protein trace shifted to a higher plateau after initial relaxation, indicating a larger receptor-level rearrangement in this induced pocket, but the ligand traces changed gradually rather than showing a stepwise escape event. In the TYSY KLEP7 10-propargyl-5, 8-dideazafolic acid system, the protein backbone remained comparatively steady, whereas the ligand-fit-on-protein trace increased during the middle of the trajectory and then partially relaxed, suggesting local pose reorientation or pocket accommodation rather than loss of ligand integrity. In the updated TYSY KLEP7 ANX-510 system, the protein trace rapidly entered a stable plateau, while both ligand traces showed early-to-middle trajectory adjustment followed by a quieter late-trajectory regime. Together, these trends support persistent but adaptive engagement of Compound 8 across human and bacterial TYMS/ThyA pocket states, with the larger RMSD changes best interpreted as receptor/pocket motion and local ligand repositioning rather than wholesale ligand dissociation.

The revised ligand-protein contact diagrams shown in **Figure 7** further support persistent but pocket-dependent engagement. In the human 10-propargyl-5, 8-dideazafolic acid model, the >40% contact set included Asn112 (61%), His256 (59%), Asp218 (65%), Glu87 (71%), Phe225 (46%), Met309 (40%), Ile307 (43%), and Lys77 (59%). In the human ANX-510 model, recurrent contacts shifted toward Glu87 (79%), Phe225 (70%), Met309 (61%), Arg78/water-mediated contacts (61–67%), Asp218 (50%), His256 (48%), and Asn112 (42%). In the bacterial TYSY KLEP7 10-propargyl-5, 8-dideazafolic acid model, His207 was retained throughout the selected contact trajectory (100%), with additional >40% contacts involving Tyr209 (48%), Leu172 (43%), Lys48 (60–62%), and Ile258 (47%). The bacterial ANX-510 model showed a similar His207 anchor (100%) together with Tyr209 (51%), Val262 (40%), Leu172 (53–65%), Lys48 (59–65%), and Ile258 (53%).

Together, the 1000 ns RMSD traces in **Figure 6** and the >40% ligand-protein contact maps in **Figure 7** support the docking-derived hypothesis that Compound 8 can remain engaged in both human and bacterial TYMS/ThyA pocket contexts while adapting its local interaction network. We interpret the simulations as supportive pose-stability evidence rather than definitive thermodynamic convergence, because the contact diagrams summarize a selected 0–100 ns trajectory window and the RMSD trend evaluation does not replace purified-enzyme binding or inhibition measurements.

### 3.7 Computer-aided synthesis planning supports a convergent route hypothesis for compound 8

**Figure 8:**
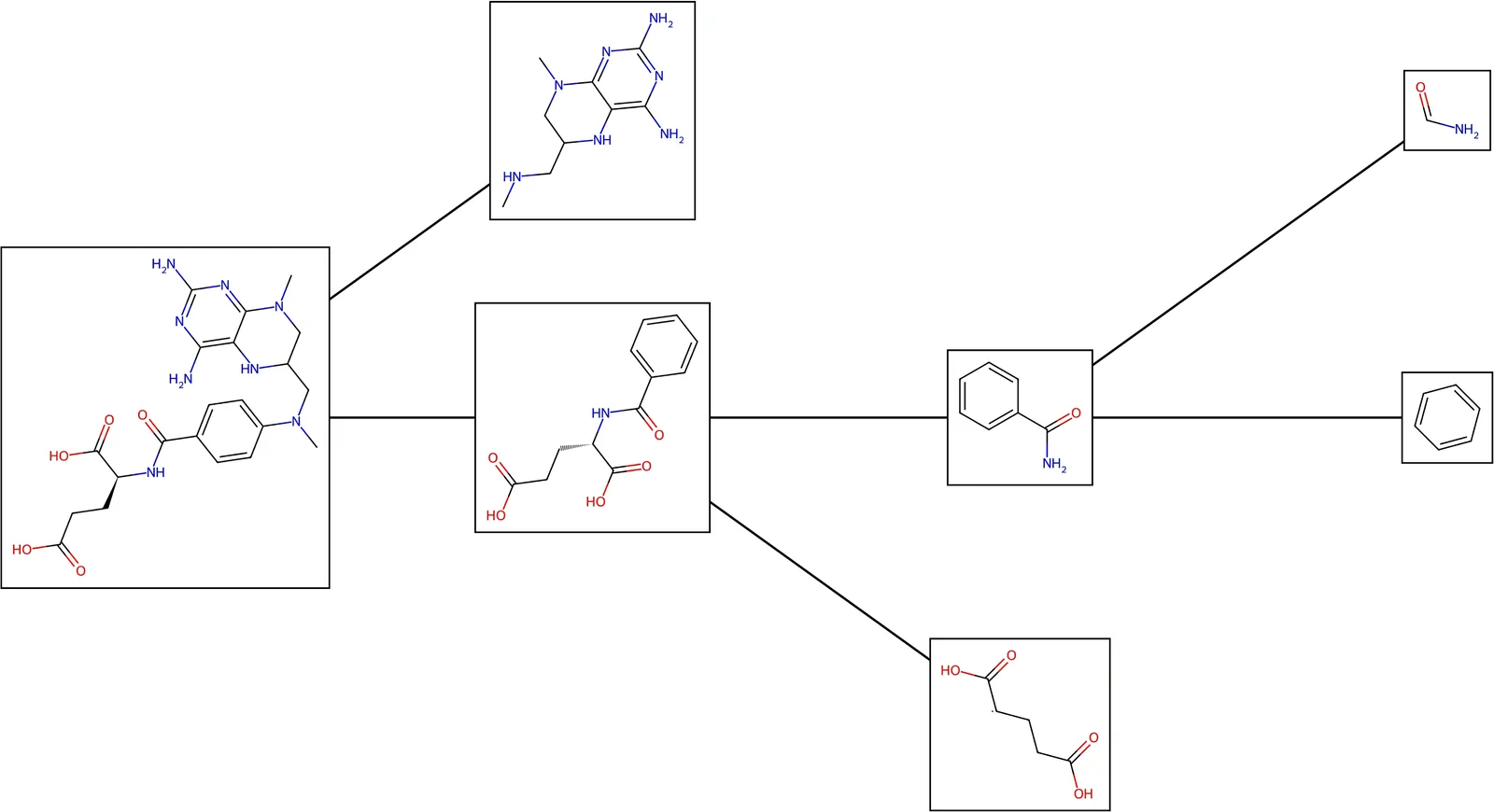
Computer-aided synthesis planning tree and principal route for Compound 8. The route is a computationally proposed synthesis-planning hypothesis derived from BRICS-style decomposition and should not be interpreted as an experimentally optimized or completed synthesis.

We performed computer-aided synthesis planning using Breaking of Retrosynthetically Interesting Chemical Substructures (BRICS) (Degen et al., 2008), which was implemented through the local RDKit BRICS module, to examine whether Compound 8 could be disconnected into chemically plausible modules (**Figure 8**; **Supplementary Figure 1**). The BRICS-derived tree is interpreted as a route-planning hypothesis rather than an experimentally optimized synthesis. The proposed forward logic contains three operations: assembly of an aromatic amide amino acid module, palladium-catalyzed C-N fragment union, and final deprotection to the dicarboxylic acid.

The dicarboxylate unit can be introduced from a protected glutamate derivative. Masking the carboxyl groups as tert-butyl or methyl esters would allow acylation of the α-amino group with an aryl benzoyl electrophile bearing a halogen or related coupling handle. This protected aryl-electrophile benzamide-glutamate intermediate could be prepared through acid chloride chemistry or standard peptide-coupling conditions such as HATU or EDC.

Fragment union can then be achieved with a fused N-heterocyclic aminomethyl primary amine through Buchwald-Hartwig amination. Typical conditions would use a palladium catalyst, phosphine ligand, and base such as cesium carbonate in dioxane or a related solvent. Late-stage N-methylation or N-formylation can complete the head-group architecture when required.

Global deprotection would regenerate the terminal dicarboxylic acid. The required building-block classes are plausible at a medicinal-chemistry planning level: protected glutamate esters and cyclopropanecarboxylate/acylating reagents are common classes; aryl electrophiles and heterocyclic amine partners are plausible catalog or short-step synthetic inputs; and standard amide-coupling, palladium-coupling, and deprotection reagents are routinely available. Vendor sourcing, reaction optimization, purification, and analytical confirmation remain necessary before experimental synthesis can be claimed.

### 3.8 Compound 8 exhibits dose-dependent anti-CRC activity with modest antibacterial potency against KLEP7

**Figure 9:**
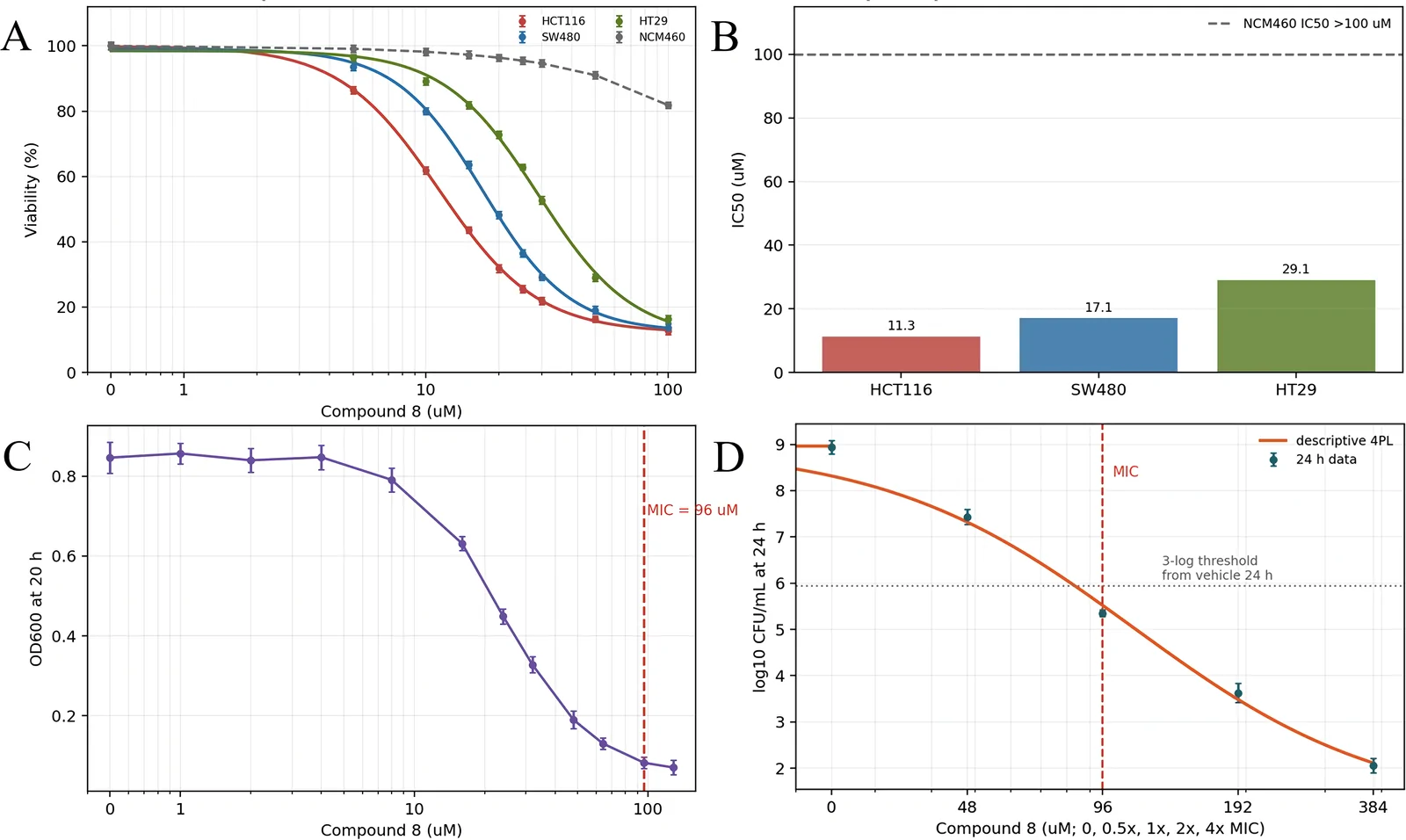
Dose-dependent effects of Compound 8 on colorectal cancer cell viability, NCM460 tolerability, MIC, and time-kill behavior against KLEP7. **(A)** Updated 0–100 µM CCK-8 dose-response curves for HCT116, SW480, HT-29, and NCM460 cells, normalized to the 0 µM control. Points show same-batch replicate-well means plus or minus SD; fitted curves are four-parameter logistic models where applicable. **(B)** IC_50_ summary for CRC cells with the NCM460 IC_50_ shown as >100 µM, supporting conservative selectivity-index lower bounds. **(C)** Broth microdilution MIC readout for KLEP7, showing the no-visible-growth threshold at 96 µM. **(D)** Semi-log 24 h time-kill dose-response for KLEP7, including the MIC marker, descriptive 4PL fit to group means, and the conventional 3-log bactericidal threshold. Same-batch replicate wells and time-kill assay replicates are interpreted as exploratory screening evidence rather than independent biological replication.

The impact of Compound 8 on cell survival was evaluated using an updated 0–100 µM CCK-8 assay across three colorectal cancer cell lines and a normal colorectal epithelial control as shown in **Figure 9A, B**. The dataset used ten same-batch replicate wells per concentration, and results are presented as the mean plus or minus the standard deviation after normalization to the corresponding vehicle control. Because these same-batch wells are not independent biological replicates, the statistical summaries describe within-assay dispersion and are interpreted as exploratory screening evidence. One-way ANOVA was used only as an omnibus assessment of concentration-associated differences within this screen.

Specific sensitivity patterns were identified through prespecified comparisons versus the control using the Welch *t*-test with Holm correction. In the updated same-batch CCK-8 dataset, HCT116 viability decreased to 61.8 ± 1.1% at 10 µM, 43.6 ± 1.0% at 15 µM, and 12.7 ± 1.2% at 100 µM. SW480 viability decreased to 48.3 ± 1.1% at 20 µM and 13.6 ± 1.1% at 100 µM. HT-29 was more tolerant at lower concentrations but decreased to 52.7 ± 1.2% at 30 µM and 16.4 ± 1.1% at 100 µM. Four-parameter logistic modeling yielded IC_50_ values of 11.30 µM for HCT116 (95% CI 11.13–11.47 µM; R^2^ = 0.9986), 17.13 µM for SW480 (95% CI 16.88–17.39 µM; R^2^ = 0.9981), and 29.06 µM for HT-29 (95% CI 28.38–29.75 µM; R^2^ = 0.9970). These confidence intervals reflect same-batch replicate-well variability rather than independent biological replication. Normal NCM460 cells retained 81.8 ± 0.9% viability at 100 µM and did not reach an IC_50_ within the 0–100 µM range, supporting preliminary tolerability in normal colonic epithelial cells at the antibacterial concentration range. Using 100 µM as the conservative lower bound for NCM460 IC_50_, the estimated selectivity-index lower bounds were SI(NCM460/HCT116) > 8.85, SI(NCM460/SW480) > 5.84, and SI(NCM460/HT-29) > 3.44. These SI values support a preliminary tumor-versus-normal-colon epithelial window, but they do not establish a complete systemic safety profile.

The antibacterial effect of Compound 8 against KLEP7 is now presented using MIC and time-kill data rather than relying on the initial endpoint CFU screen. The original endpoint CFU screen showed only a shallow, high-variance reduction at 100 µM, approximately 0.74 log_10_ CFU/mL relative to vehicle, and is therefore interpreted descriptively rather than used as formal potency evidence. Broth microdilution placed the MIC at 96 µM: visible growth persisted through 64 µM (OD_600_ = 0.131 ± 0.015), whereas 96 and 128 µM showed no visible growth with OD_600_ values of 0.082 ± 0.015 and 0.070 ± 0.018, respectively. Time-kill analysis showed that vehicle cultures increased by +3.12 log_10_ CFU/mL at 24 h, whereas 0.5× MIC increased by +1.57 log_10_ CFU/mL, 1× MIC decreased by -0.49 log_10_ CFU/mL, 2× MIC decreased by -2.19 log_10_ CFU/mL, and 4× MIC decreased by -3.74 log_10_ CFU/mL. Revised **Figure 9D** plots the 24 h time-kill burdens on a semi-log concentration axis with a descriptive four-parameter logistic fit to the group means (EC_50_-like transition concentration 112.77 µM; R^2^ = 0.9979). This fitted transition is not an MIC or MBC estimate. Thus, Compound 8 was primarily growth-suppressive around the MIC, showed sub-bactericidal killing at 2× MIC, and reached the conventional bactericidal threshold only at 4× MIC after 24 h. A separate MBC value was not determined, and only one *K. pneumoniae* strain was tested. Overall, these data support modest antibacterial potency rather than clinically meaningful antibacterial activity.

### 3.9 qPCR and crude-lysate TS activity support *TYMS*/*thyA* target-axis engagement

To address whether the phenotypic effects are linked to the proposed target axis, *TYMS*/*thyA* transcript suppression and crude-lysate TS activity assays were added. In HCT116 cells, all three *TYMS* siRNAs reduced *TYMS* mRNA relative to the non-targeting siRNA control. siTYMS-H2 was the strongest design, with relative *TYMS* expression of 0.171 ± 0.030 and 82.9% suppression; siTYMS-H1 and siTYMS-H3 produced 75.4% and 68.4% suppression, respectively. In *K. pneumoniae* ATCC 700721, *thyA* transcript suppression was also observed, with AS-*thyA*-K2 giving the strongest reduction (relative expression 0.343 ± 0.129; 65.7% suppression), followed by AS-*thyA*-K1 (49.6%) and AS-*thyA*-K3 (39.8%).

Compound 8 inhibited TS activity in both crude-lysate systems in a dose-dependent manner. In HCT116 lysate, Compound 8 showed an apparent AC_50_ of 7.8 µM, with residual activity of 58.2 ± 7.6%, 39.6 ± 7.2%, and 28.4 ± 6.5% at 10, 30, and 100 µM, respectively. In *K. pneumoniae* lysate, Compound 8 showed an apparent AC_50_ of 13.2 µM, with residual activity of 69.3 ± 8.3%, 49.2 ± 8.5%, and 35.9 ± 7.2% at the same doses. Under activation-compatible conditions, 5-FU produced stronger apparent TS-pathway inhibition, with AC_50_ values of 3.6 µM in HCT116 lysate and 6.8 µM in *K. pneumoniae* lysate. FdUMP positive controls confirmed assay sensitivity to canonical TS inhibition, with apparent AC_50_ values of 0.045 and 0.075 µM in the human and bacterial lysates, respectively. These data support TS-axis engagement by Compound 8 while also showing that its apparent potency is weaker than the 5-FU/FdUMP control axis. Importantly, this target-axis support is distinct from whole-cell antibacterial potency, which remains modest in the current endpoint CFU assay.

## 4 Discussion

The current study is positioned within established TYMS and antifolate pharmacology rather than as a new conceptual discovery of folate-pathway conservation. TYMS-directed chemotherapy is a standard component of CRC treatment, and CRC patients may also experience compromised mucosal integrity, suppressed immunity, and opportunistic infections such as *Klebsiella pneumoniae* (Geng, 2024; Guijarro et al., 2023). This clinical background motivated an early lead-discovery question: whether structural conservation of the thymidylate synthase/ThyA enzyme family could be used to prioritize a single antifolate-like chemical scaffold for dual target-axis engagement against human and bacterial enzymes. The study does not model secondary infection or claim clinical treatment readiness. Our integrated approach combining sequence analysis, AlphaFold3-derived complex modeling, multi-context computational screening, and preliminary biochemical validation identifies a transferable binding framework, but it does not claim that exploiting folate-pathway conservation is itself new.

A fundamental requirement for any dual-target strategy is a conserved catalytic core capable of supporting shared anchoring interactions (Ye et al., 2023). For this reason, the structural comparison was built from matched AlphaFold3/Chai-1 human and KLEP7 models across equivalent ligand states, because a complete matched experimental human/KLEP7 ligand-state structure set was not available. Our findings support this criterion: the catalytic domain maintains high residue identity and physicochemical similarity despite species-specific features such as the flexible N-terminal extension in the human enzyme (Borkakoti et al., 2025). The binding pocket is even more conserved than the catalytic-domain average, and the catalytic cysteine pair, human Cys195 and KLEP7 Cys146, aligns in the same functional region. The low Cα RMSDs between the human predicted models and human TYMS crystal structures further support use of the modeled human active-site geometry for comparative analysis, while model-confidence scores indicate that the pocket regions are more reliable than flexible terminal or loop segments. This pattern supports the use of direct antifolate-like references as templates for a shared lead-discovery axis, while also emphasizing that protein-fold conservation alone does not establish equivalent cellular potency, permeability, or selectivity.

The feasibility of this transferable framework was evaluated by modeling established human TYMS ligands in complex with both enzymes. This analysis should be read in the context of prior antifolate literature: trimethoprim and pyrimethamine illustrate antibacterial and antiprotozoal DHFR-directed folate-pathway pharmacology, whereas raltitrexed, pemetrexed, 10-propargyl-5, 8-dideazafolic acid, and prior TS/DHFR dual-inhibitor studies provide closer precedent for TS-directed or multitarget antifolate design (Kompis et al., 2005; Nzila, 2006; Jackman and Calvert, 1995; Zhou et al., 2013; Adjei, 2000; Gangjee et al., 2001; Arooj et al., 2013). The observation that most direct antifolate binders maintain consistent pocket occupancy and orientation across-species provides a practical justification for using ligand-induced templates as a foundation for generative AI design (Newstead, 2022). In contrast, the variable positioning observed for Capecitabine reflects its indirect mechanism as a prodrug, reinforcing the conclusion that direct enzyme inhibitors are more suitable starting points for structure-based TYMS/ThyA discovery (Jacobs et al., 2019).

By leveraging these transferable templates, we implemented a stringent four context filtering process that integrated species origin and induced pocket states. The convergence of results suggests that cross-species compatibility is an emergent feature of specific molecular geometries that remain stable under pocket perturbation (Allard et al., 2025). The inclusion of ligand strain energy and MM-GBSA estimates was useful for distinguishing candidates with favorable docking scores from those with unrealistic conformational penalties (Genheden and Ryde, 2015). However, the MM-GBSA calculations used a VSGB implicit-solvent model and therefore do not explicitly quantify crystallographic or dynamic water-mediated hydrogen bonds. This limitation is important for TYMS inhibitors. For this reason, MM-GBSA values are interpreted as relative rescoring metrics rather than absolute affinity estimates, and explicit-solvent MD contact analysis was used to document frequent water-bridge contacts for Compound 8.

Compound 8 emerged from this prioritized set as an antifolate-like cross-species TYMS-axis lead with a binding logic that supports engagement of conserved human and bacterial pocket features. The scaffold is structurally related to classical TS antifolates through its heteroaromatic head, para-benzoyl linker, and acidic dicarboxylate tail, and is therefore not presented as an entirely novel antifolate chemotype. Its distinguishing feature in this study is the modeled head/linker plasticity that allows conserved tail anchoring while redistributing hydrogen-bond and backbone-contact networks across human TYMS and bacterial TYSY KLEP7 pocket contexts. This adaptability makes the molecule a useful starting point for target-axis validation, but the current bacterial phenotype indicates that antibacterial potency optimization is required before any translational antibacterial framing.

When placed in the context of existing antifolate and TYMS therapies, Compound 8 does not currently offer a demonstrated clinical advantage over established drugs or investigational dual-activity antifolates. Existing agents have stronger pharmacological, clinical, and medicinal-chemistry validation. The possible value of Compound 8 is instead scaffold-level and hypothesis-generating: it retains conserved TYMS/ThyA acidic-tail anchoring while allowing adaptable head/linker contacts that may guide future analog design. The added microbiological assays provide necessary context for this limited claim: the MIC against *K. pneumoniae* ATCC 700721/MGH 78578 was 96 µM, and time-kill analysis reached the conventional bactericidal threshold only at 4× MIC after 24 h. These data answer the bacteriostatic-versus-bactericidal question for this strain and assay condition, but they also reinforce that whole-cell antibacterial potency is weak to modest. A separate MBC value and resistant clinical-isolate panel were not obtained. Thus, the present data do not support Compound 8 as a clinically meaningful antibacterial agent. Instead, Compound 8 should be viewed as a TYMS-axis lead with anticancer activity and modest antibacterial coactivity that requires targeted optimization. The observed sensitivity gradient across different cancer cell lines reflects the influence of cellular context, such as nucleotide salvage and enzyme expression, on therapeutic response.

The modular and convergent synthetic route identified for Compound 8 provides a realistic platform for future structural optimization. This flexibility is essential for tuning properties that govern exposure and target engagement in both human and bacterial compartments, such as cellular uptake and host selectivity. The calculated descriptors for Compound 8 provide useful ADME context: MW 319.31, cLogP -1.80, TPSA 148.54 Å^2^, HBD 4, HBA 8, six rotatable bonds, QED 0.416, and no maximum structural alert in the prioritization table. The acidic dicarboxylate motif, low cLogP, and high TPSA suggest a likely passive-permeability liability despite favorable target-axis activity. Therefore, these data should be interpreted as early developability context rather than a completed ADME profile. The qPCR and crude-lysate TS activity assays strengthen the biological link between the computationally prioritized scaffold and the *TYMS*/*thyA* enzymatic axis. Future antibacterial optimization will focus on improving bacterial exposure and whole-cell potency while retaining TYSY KLEP7 anchoring interactions, including tuning the acidic-tail/dicarboxylate ionization state, exploring ester/prodrug or bioisosteric masking strategies, balancing polar and hydrophobic substituents for Gram-negative uptake, reducing potential efflux liability, and preserving the Lys259/folate-groove anchoring motif. Optimized analogs should then be evaluated by standardized MIC, MBC, and time-kill assays, together with a broader *K. pneumoniae* panel including antibiotic-resistant clinical isolates and experimental ADME assays such as PAMPA or Caco-2/MDCK permeability and microsomal stability.

The new target-axis assays also clarify the current evidentiary boundary. TYMS itself is already a well-established CRC therapeutic target, supported by fluoropyrimidine- and antifolate-based therapeutic literature cited above; therefore, this study is not intended to re-prove the general relevance of TYMS in CRC. The added siTYMS and *thyA* transcript-suppression data verify that the target axis can be experimentally modulated in the human and bacterial systems, and the crude-lysate activity assays show that Compound 8 reduces TS activity in both biological matrices. Nevertheless, these are apparent crude-lysate AC_50_ values rather than purified recombinant-enzyme IC_50_ measurements. Purified human TYMS and TYSY KLEP7 assays, intracellular dTTP depletion measurements, and bacterial pathway-rescue experiments would further define compound-specific target engagement. TYMS knockdown or overexpression rescue in CRC cells would further strengthen the cellular target-engagement evidence, but it is not required to establish TYMS as a CRC-relevant target. Statistically, the updated CCK-8 dataset and original endpoint CFU screen should still be regarded as same-batch or technical-replicate screening assays because n = 10 reflects replicate wells or plate-count measurements rather than independent experiments performed on different days or passages. Independent biological repeats are required before drawing stronger inference from the IC_50_ estimates, CCK-8 effect sizes, or endpoint CFU comparisons. The updated NCM460 data support preliminary normal-colonic epithelial tolerability at 100 µM and improved SI lower-bound estimates, but a single normal colonic line is insufficient to establish systemic safety. Non-colorectal normal human cells, such as HEK293 cells or primary fibroblasts, and broader normal-human-cell cytotoxicity testing at antibacterial concentration ranges remain necessary. The added MIC and time-kill assays contextualize the bacterial phenotype but do not eliminate the potency limitation: bactericidal activity was observed only at 4× MIC, MBC was not separately determined, and additional *K. pneumoniae* strains, including antibiotic-resistant clinical isolates, remain necessary before any stronger antibacterial translational claim can be made.

## 5 Conclusion

This study presents an AI-driven, structure-guided framework for prioritizing candidate cross-species ligands against human thymidylate synthase and the bacterial homolog TYSY KLEP7. The work is positioned within established antifolate pharmacology: its contribution is not the general concept of folate-pathway conservation or dual antifolate activity, but a matched structural prioritization workflow with preliminary target-axis validation. By integrating AF3/Chai-1 modeling, crystal-structure cross-validation, docking, ligand strain energy, MM-GBSA rescoring, and explicit-solvent MD, we identified Compound 8 as an early antifolate-like lead scaffold. The ligand is predicted to preserve an acidic-tail anchoring motif while redistributing head/linker contacts across human and bacterial pocket states. Phenotypic assays showed dose-dependent colorectal cancer cell inhibition and modest antibacterial activity against *K. pneumoniae*. Target-axis assays supported human and bacterial TS engagement, with apparent crude-lysate AC_50_ values of 7.8 and 13.2 µM, respectively; MIC and time-kill assays placed the MIC at 96 µM and showed bactericidal activity only at 4× MIC after 24 h. Calculated descriptors indicate a likely permeability liability associated with the acidic dicarboxylate scaffold. Further work should include purified-enzyme IC_50_ measurements, intracellular pathway-rescue studies, biological replication of cell and bacterial assays, broader normal-cell and strain-panel testing, experimental ADME/permeability studies, MBC determination, and experimental synthesis. Overall, this work provides a hypothesis-generating computational-to-phenotype lead-discovery paradigm for future optimized anticancer and antibacterial lead development.

## Data Availability

The raw data supporting the conclusions of this article will be made available by the authors, without undue reservation.
