# Supplementary figures and images for: Artificial intelligence-guided discovery of a lead compound with antifolate-like activity against bacterial and human thymidylate synthases
Source: Front Cell Infect Microbiol. 2026 Jun 26;16:1816809. doi: 10.3389/fcimb.2026.1816809 (PMC13392931; doi:10.3389/fcimb.2026.1816809)

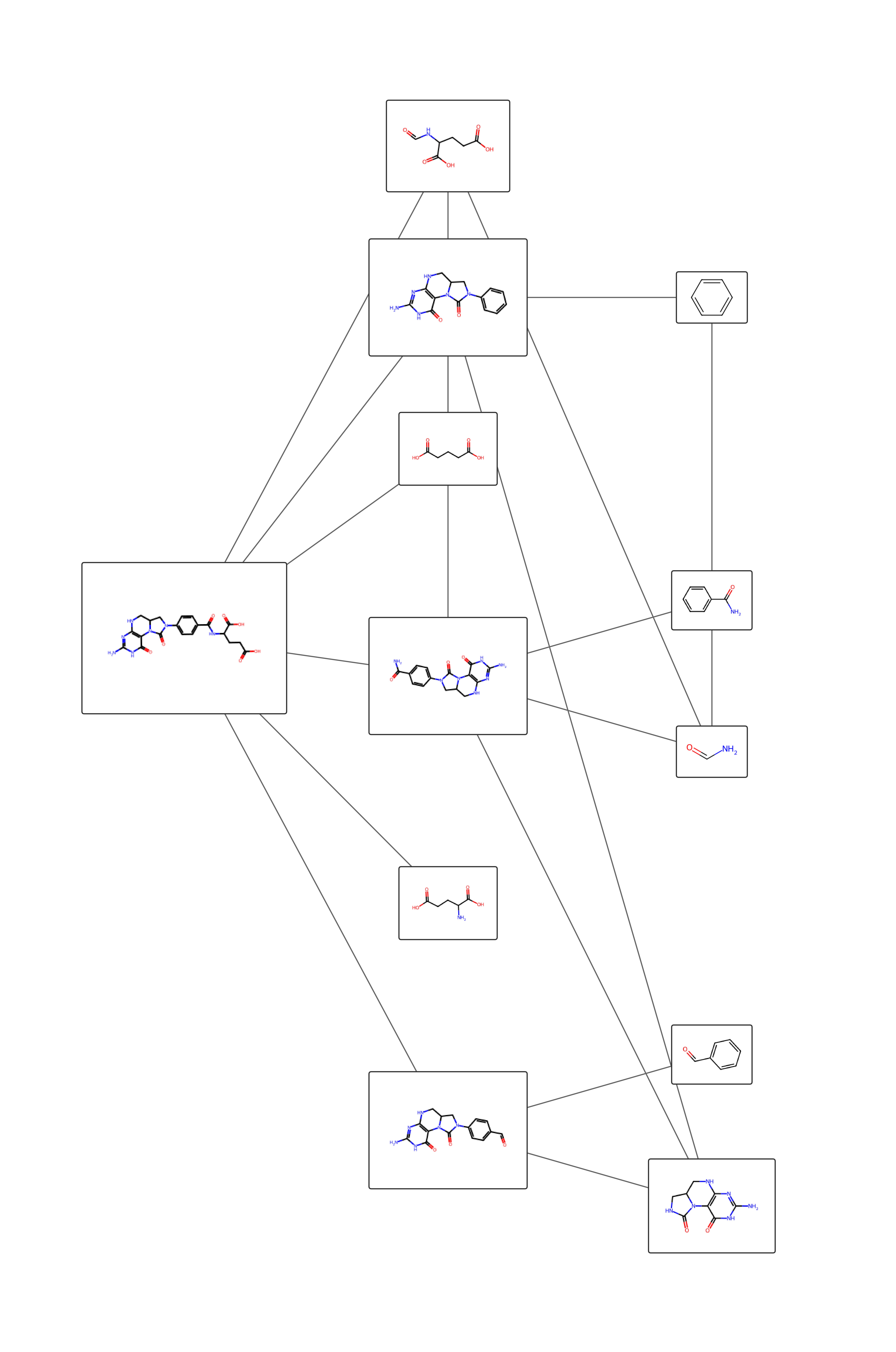

Supplement: Supplementary file 2 [file Image1.jpeg]
